# A multi-GPU accelerated virtual-reality interaction simulation framework

**DOI:** 10.1371/journal.pone.0214852

**Published:** 2019-04-11

**Authors:** Xuqiang Shao, Weifeng Xu, Lina Lin, Fengquan Zhang

**Affiliations:** 1 School of Control and Computer Engineering, North China Electric Power University, Baoding, Hebei, China; 2 92524 troops, Ningbo, Zhejiang, China; 3 School of Computer Science, North China University of Technology, Beijing, China; Central University of Finanace and Economics, CHINA

## Abstract

In this paper, we put forward a real-time multiple GPUs (multi-GPU) accelerated virtual-reality interaction simulation framework where the reconstructed objects from camera images interact with virtual deformable objects. Firstly, based on an extended voxel-based visual hull (VbVH) algorithm, we design an image-based 3D reconstruction platform for real objects. Then, an improved hybrid deformation model, which couples the geometry constrained fast lattice shape matching method (FLSM) and total Lagrangian explicit dynamics (TLED) algorithm, is proposed to achieve efficient and stable simulation of the virtual objects’ elastic deformations. Finally, one-way virtual-reality interactions including soft tissues’ virtual cutting with bleeding effects are successfully simulated. Moreover, with the purpose of significantly improving the computational efficiency of each time step, we propose an entire multi-GPU implementation method of the framework using compute unified device architecture (CUDA). The experiment results demonstrate that our multi-GPU accelerated virtual-reality interaction framework achieves real-time performance under the moderate calculation scale, which is a new effective 3D interaction technique for virtual reality applications.

## Introduction

Natural and real-time 3D interaction with computer-generated virtual environment is an important research topic of virtual reality and augmented reality technologies, as it increases the participant’s sense of immersion. Up to now, there have been many practical application examples of 3D interaction framework based on the haptic feedback devices, especially in the more and more commonly used virtual surgery area. For example, Bro-Nielsen et al. [1] developed a virtual surgical simulator for training the removal of a shattered kidney by open surgery, which allows the users to probe and cut the virtual soft tissues by using the SenSable Technologies Phantom haptic feedback device. By supporting haptic feedbacks through Phantom Omni devices, Webster et al. [2] proposed a realistic, easy to operate, and economical prototype of haptic suturing simulator to train basic suturing operations for simple wound closure. To support complex virtual cutting interactions, Bielser et al. [3] designed a realistic open surgery trainer which could basically model various interactions of the elastic soft tissue and the virtual scalpel or hook controlled by the force feedback device. Furthermore, to achieve more realistic virtual cutting interactions with bleeding effects, Rianto et al. [4] incorporated GPU accelerated fluid dynamic simulation into a heart surgery simulator exploiting haptic feedback devices. Thus when the surgical knife cut the heart muscle surface in a certain thickness, the blood flowing on the surface of a beating heart is realistically modeled in real time.

Although the above 3D interactions with virtual environments are realistic and immersive, haptic feedback devices are too cumbersome and expensive for common users. With the rapid development of image-based 3D reconstruction technology, camera-based virtual-reality interaction has become a new type of effective 3D interaction technique for virtual reality applications. Up to now, a variety of image-based 3D reconstruction algorithms such as structure from stereo [5], structure from motion [6], shape from shading [7], and shape from silhouettes [8], have been developed. Due to computing the intersection volume of all viewing cones, the 3D reconstruction method from silhouettes is also regarded as visual hull method. Visual hulls are divided into two categories: surface-based visual hull (SbVH) [8] and voxel-based visual hull (VbVH) [9]. SbVH method, which generates 3D polyhedral intersections, is relatively inefficient for the special-case problems, and suffers from numerical instabilities. VbVH method, by contrast, is not susceptible to numerical difficulties that may arise in the SbVH method, and suitable for being accelerated by CUDA. Based on VbVH method, INRIA developed a platform called Grimage [10] which provides the realistic simulation of the interactions of reconstructed objects and computer-generated virtual objects. When any real objects are put into the interaction space, their reconstructed 3D models are instantaneously computed and placed into a virtual environment to interact with computer-generated rigid or elastic objects. However, VbVH whose reconstruction accuracy depends on the number of cameras is only the approximation of the real object. For each point of the image, a time-of-flight (TOF) camera measures the time of flight of a light signal between the camera and the subject. Through refining the reconstructed VbVH by the depth map from a TOF camera, Wang et al. [11] presented a real-time reconstruction platform implemented on the GPU. This platform supports the reconstructed person model pushing, catching and squeezing the virtual rigid objects. Although VBVH-based virtual-reality interaction technology has achieved natural and real-time 3D interaction experiences for the users, in our opinion, there are still several problems to be solved:

1. The 3D reconstruction precision of VbVH method is not high enough due to the occlusion between the models, which leads to unnatural virtual-reality interactions for the users.
2. The supported types of virtual-reality interactions are limited, because the virtual objects are rigid and do not support topology changes.
3. For complex virtual-reality interaction scenarios, the time-consuming computation loads of each time step limit its real-time or interactive applications.

In order to solve the above problems to improve the interaction experiences, based on our previous works on hybrid deformation model for virtual cutting [12], fluid-solid coupling [13] and visual hull reconstruction [14], we develop a type of image-based virtual-reality interaction framework implemented on multiple GPUs using CUDA, which supports more types of interactions including virtual cutting and achieves higher computational efficiency compared to other similar methods. Specifically, compared to other camera-based virtual-reality interaction framework, the main contributions of this paper are summarized as follows:

1. Based on an extended VbVH algorithm, we develop a more precise image-based 3D reconstruction platform for real objects.
2. An improved hybrid deformation model, which couples the geometry constrained fast lattice shape matching method (FLSM) and total Lagrangian explicit dynamics (TLED) algorithm, is proposed to achieve efficient and stable simulation of virtual objects’ elastic deformations.
3. One-way virtual-reality interactions including soft tissues’ virtual cutting with bleeding effects are successfully simulated.
4. With the purpose of significantly improving the computational efficiency of each time step, we put forward an entire multi-GPU implementation method of the framework using CUDA.

The experiment results demonstrate that our virtual-reality interaction simulation framework achieves 25 *FPS* under the moderate calculation scale, which provides a new effective 3D interaction technique for real-time virtual reality applications.

## Materials and methods

### System architecture

**Fig 1:**
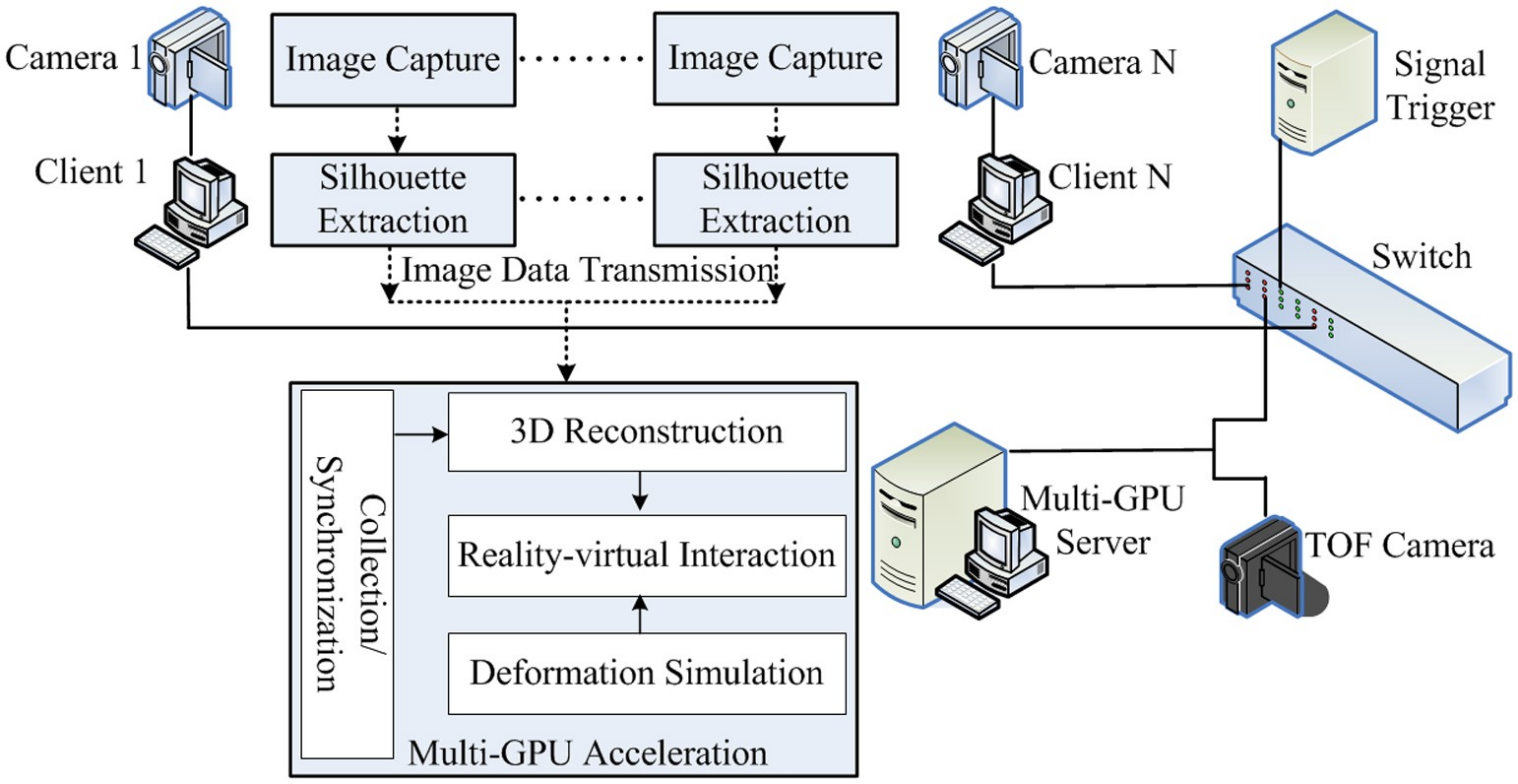
System architecture.

**Fig 2:**
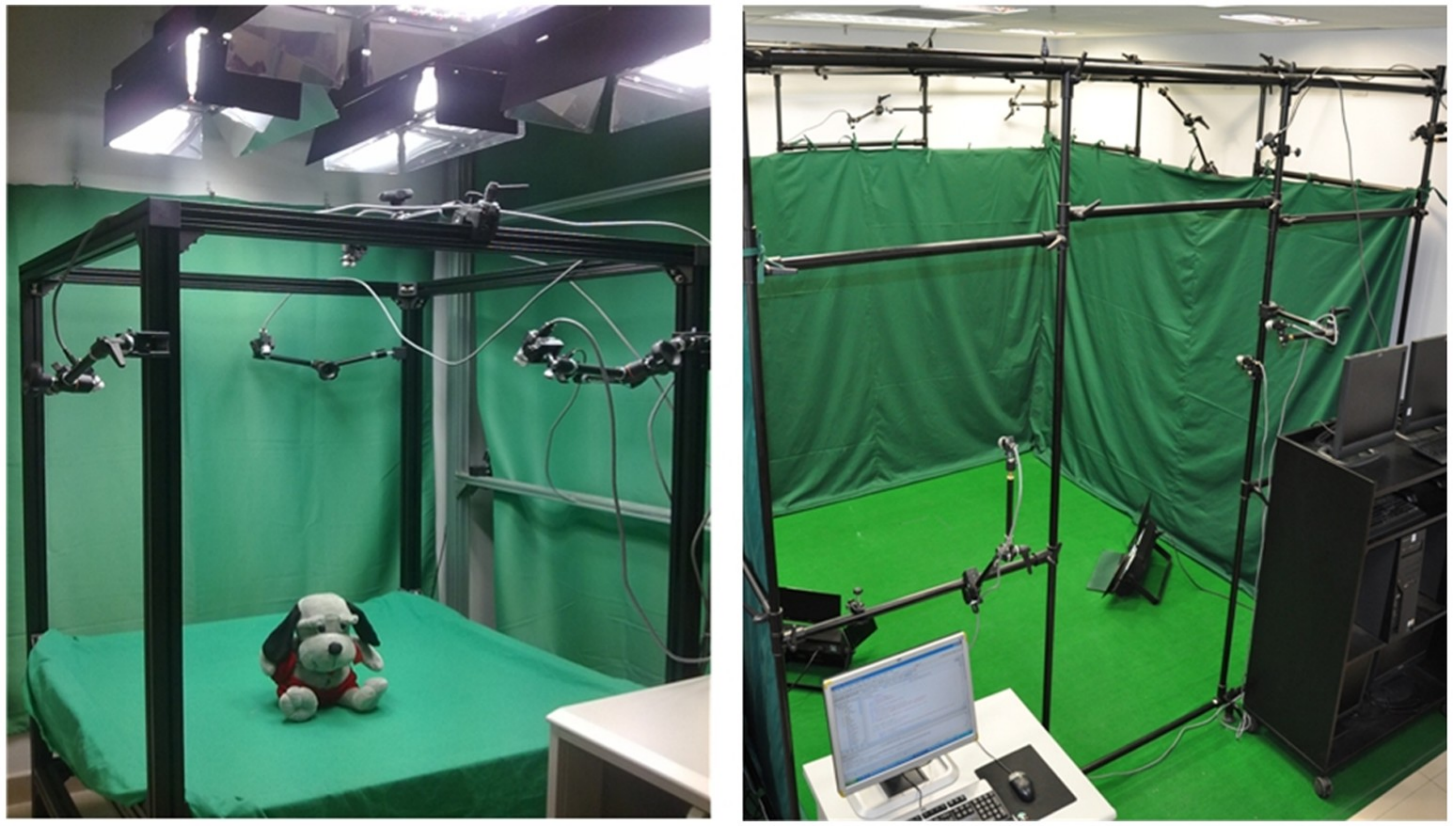
Two multi-camera acquisition platforms of different sizes. 1m×1m×1m(left) and 3m×3m×3m(right).

Based on the previous research work on image-based 3D reconstruction [11, 12, 14, 15], we design a new multi-GPU accelerated virtual-reality interaction simulation framework. The system architecture of our virtual-reality interaction framework is shown as Fig 1: In order to obtain the images of objects to be reconstructed from different perspectives, as shown in Fig 2, 8 high-speed optical cameras and 1 TOF depth camera are placed on a 1*m*×1*m*×1*m* or 3*m*×3*m*×3*m* sized frame in different positions. Each camera is connected to a single PC. Before the system runs, the geometric calibration of all these cameras are implemented by using Zhang’s method [16] to get the intrinsic and extrinsic camera parameters, and the depth calibration of TOF camera is implemented by using Wang’s method [11] which constructs lookup table to correct system error. The synchronization of multiple heterogeneous cameras is realized by using the external triggering mechanism of [11] which sends both high and low level signals to the camera signal interface alternately. As shown in Fig 1, the clients, the server, signal trigger, and the TOF camera are connected together by a switch. The Client/Server mode is adopted to construct the system framework. When acquiring the images from their connected cameras, these PCs locally execute the background subtraction method [17] to extract the silhouette of the objects to be reconstructed. Once the silhouettes are computed, each one is sent to the server which is a high performance graphic workstation with multiple GPUs. The multi-GPU server is in charge of parallel 3D reconstruction of real objects, virtual objects’ deformation simulation and virtual-reality interaction.

### Ethics statement

The individual in this manuscript has given written informed consent (as outlined in PLOS consent form) to publish these case details.

### 3D reconstruction of real objects

**Fig 3:**
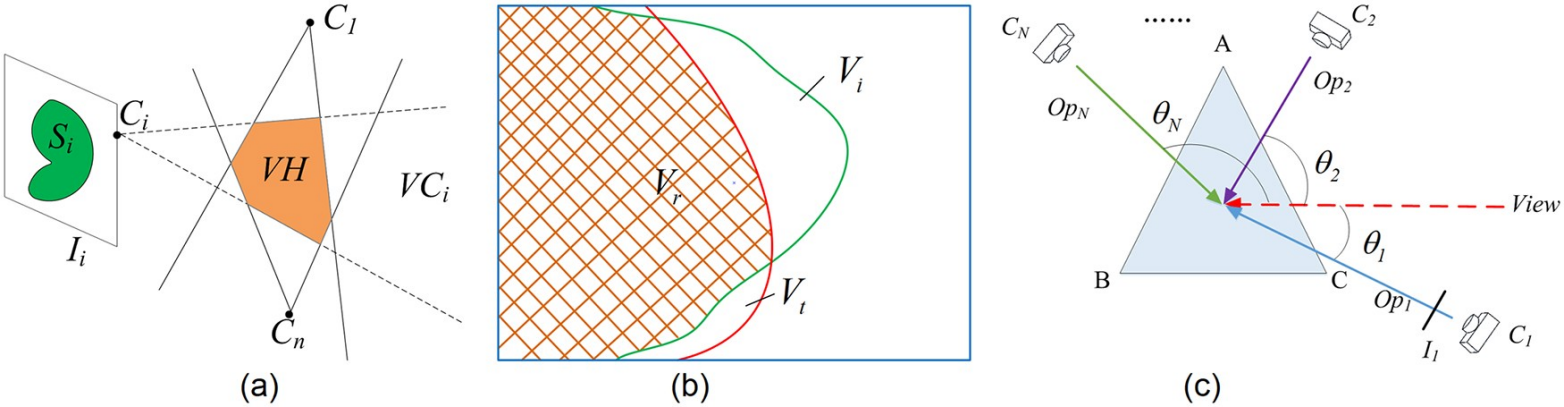
Extended VbVH-based 3D reconstruction. (a) Visual hull computation; (b) Surface corrected by depth map data; (c) Texture mapping.

To reconstruct more accurate objects from the captured images, we put forward an extended VbVH method [15] for 3D reconstruction which exploits the data of TOF depth camera to correct the triangulated surface generated by marching cubes (MC) algorithm. There are three modules for our extended VbVH 3D reconstruction method: (1) visual hull computation, means intersection testing of viewing cones to determine the status of each voxel (Fig 3(a)); (2) triangulation corrected by depth data, means combining depth map data and MC algorithm [18] to extract the triangular mesh surface of the object from the visual hull (Fig 3(b)); (3) texture mapping, means determining the texture source of each triangle for realistic surface rendering (Fig 3(c)).

In our method, the principle of visual hull computation is: (1) in the preprocessing stage, the acquisition space is divided into a series of independent voxels with a certain size. For all voxels, we create a mapping which projects their three-tuple indices as the three dimensional coordinates in the reference framework of TOF camera; (2) in the runtime stage, the occupancy status of each voxel is computed to estimate the reconstructed shape of the real object. Each voxel has one and only one status which belongs to the status set {***Inside***, ***Outside***, ***Uncertain***}. We determine the status of each voxel according to the following rules.

If the projection of a voxel on each imaging plane lies completely inside the corresponding silhouette, the status of the voxel is defined as ***Inside***;

If the projection of a voxel on each imaging plane lies completely outside the corresponding silhouette, the status of the voxel is defined as ***Outside***;

Otherwise, it means that the status of the voxel can not be defined under the current computational precision, so we define the status of the voxel as ***Uncertain***.

For the object to be reconstructed, all the voxels whose statuses marked as ***Inside*** constitute its inside, and all the voxels whose statuses marked as ***Uncertain*** contain its boundary. However, the voxel-based volumetric representation of the visual hull can not exactly reflect the boundary information of the object to be reconstructed. As shown in Fig 3(b), based on the method of [11], we combine depth map data and MC method to extract the triangular mesh surface of the object from the visual hull. *V*_*i*_ is the reconstruction result of standard VbVH method, and is combined with the 2.5 dimensional depth map *V*_*t*_ to generate the more accurate reconstructed triangular surface *V*_*r*_ = *V*_*i*_ ⋂ *V*_*t*_. Specifically, for a node *i* of the voxel marked as ***Inside*** or ***Uncertain***, we compute a difference value *D*_*i*_ between its distance to TOF camera *A*_*i*_ and its depth value *E*_*i*_ in depth map:

$$D_{i}=A_{i}-E_{i}$$ (1)

**Fig 4:**
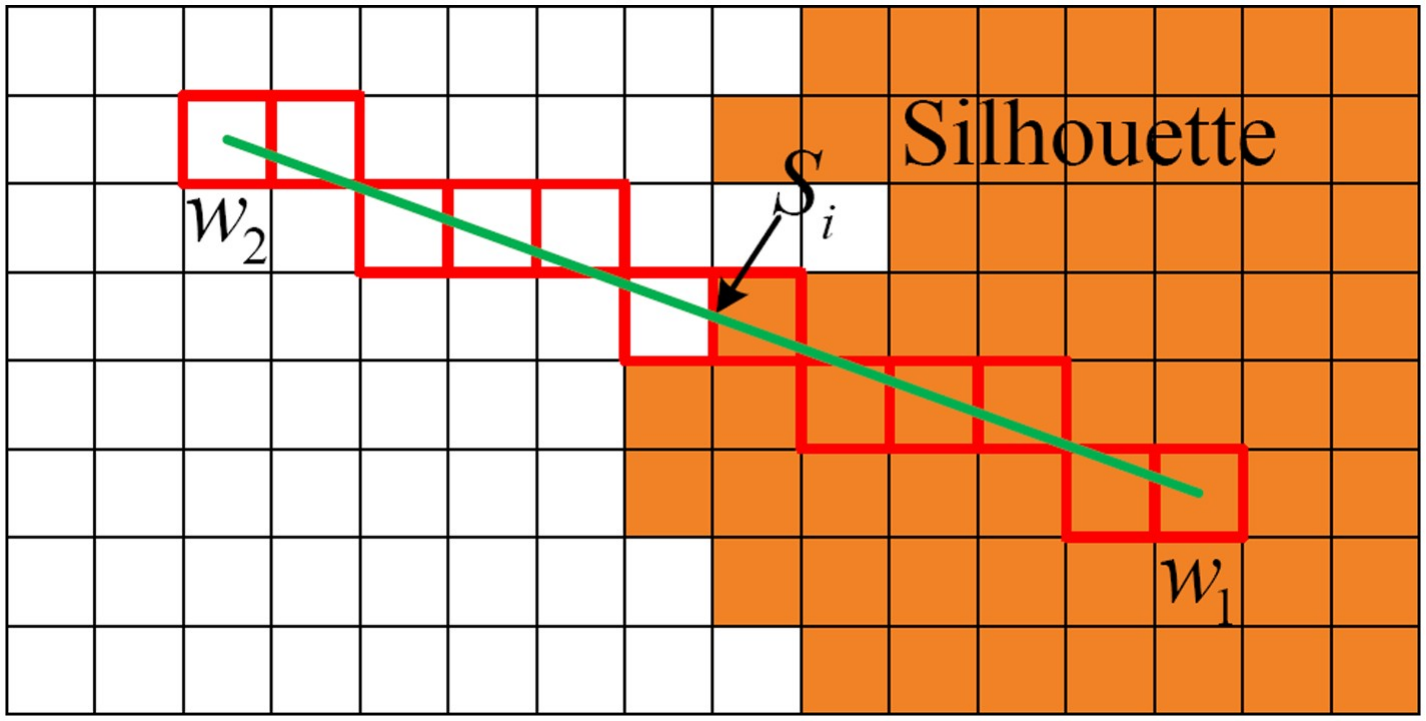
Compute the intersection point of the silhouette and the projection line.

Then according to the difference values of all nodes of each voxel, we compute the positions of vertices of the extracted triangular mesh. For a voxel, if there exists *D*_*i*_ ⋅ *D*_*j*_ < 0 for node *i* and node *j*, the position of the vertex generated by combining MC method and depth map data is:

$$Pos_{ij}=\frac{P_{i}+P_{j}\times scale}{1+scale},$$ (2)

where **P** denotes the node’s position, $scale=\left|\frac{D_{i}}{D_{j}}\right|$. For an arbitrary node *k* of a voxel, if *D*_*k*_ > 0, we exploit the silhouette information to correct the surface mesh extracted by MC method. As shown in Fig 4, the projection *w*_1_ of node 1 on each imaging plane lies completely inside the silhouette, and there exists a projection *w*_2_ of node 2 on an imaging plane lying outside the silhouette. In this case, on the imaging plane *plane*_*i*_, we use the traversal algorithm like Bresenham algorithm [19] to calculate the intersection point *S*_*i*_ of the silhouette and the line *w*_1_*w*_2_. By computing the back projection of *S*_*i*_, we obtain the vertex of the extracted triangular surface.

The polyhedral mesh extracted by MC algorithm needs to be textured for rendering. As shown in Fig 3(c), we adopt a view-dependent texture mapping algorithm which can obtain a good visual quality from the viewpoints of the cameras [14]. Take one triangular surface Δ*ABC* for example, when rendering in current viewpoint *View*, we choose the closest camera direction as the texture source. In Fig 3(c), we can see the relationship between the desired viewpoint and the cameras’ directions. Suppose that there are *N* cameras. Obviously if the optical axis of the camera is the same with the viewpoint direction, the projection on this camera’s image plane is the most clear and suitable texture it can choose. Therefore, we just need to compute the angle between *View* and each optical axis of the camera *Op*_*j*_. Denote the angle between *View* and *Op*_*j*_ as *θ*_*j*_, *j* = 1, 2, ⋯, *N*. If *θ*_1_ is the smallest among {*θ*_*j*_}, we project the three vertices of Δ*ABC* on imaging plane *I*_1_, and select the area surrounded by the projected triangle as the final texture.

### Virtual deformation simulation

We put forward an improved version of the hybrid deformation model [12, 20] to simulate the large deformation of virtual objects. As shown in Fig 4, this deformation model divides the body of deformable object into the geometrically-based deformation region (colored green) and the physically-based deformation region (colored blue). The geometrically-based deformation region which need not be physically accurate is simulated by FLSM method [21]. And we adopt the TLED method [22] to simulate the physically-based deformation region which is derived from continuum mechanics and thus is physically accurate. When we exploit the hybrid model to stably simulate the large deformation of virtual objects, the key is how to compute the deformation of calculation nodes from different deformation models, especially the interfacial calculation nodes (colored red) between two models. To achieve more stable deformation simulation, compared to the previous method [12, 20] which computes the deformations of FLSM, international and TLED nodes successively, our hybrid deformation model adopts a new method for computing three different nodes’ displacements in another order. Especially for the interfacial calculation nodes, we adopt a stable position-constrained method to compute their displacements, rather than the force-based method [12, 20] which needs to adjust a user-defined coefficient to get the desirable deformation. The calculating process of the improved hybrid model is detailed as follows:

#### Deformation computation of TLED nodes

**Fig 5:**
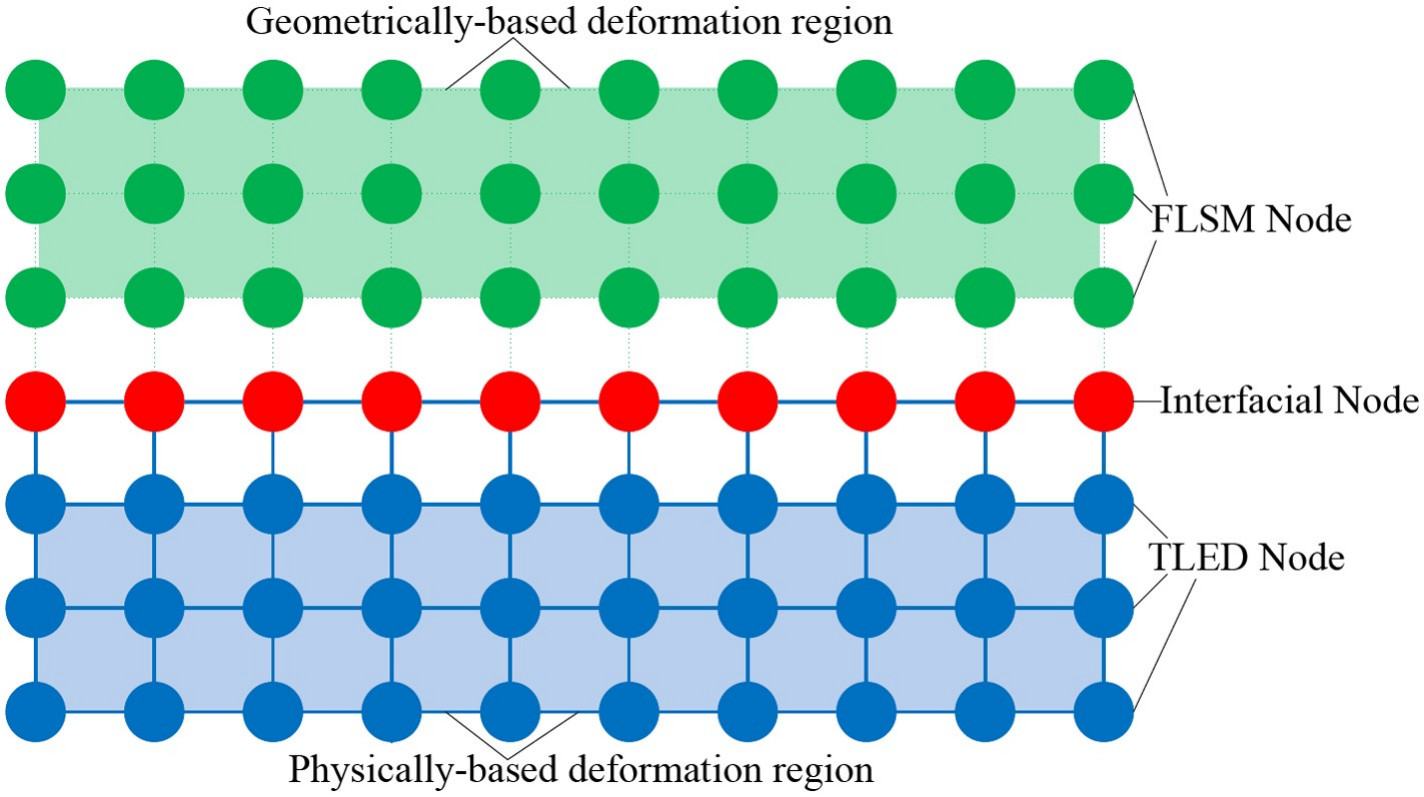
The hybrid deformation model. Geometrically-based deformation region (colored green) and physically-based deformation region (colored blue).

As shown in Fig 5, the interfacial calculation nodes belong to the finite elements situated near the interface, so we compute their deformations using TLED method by treating them as the TLED nodes. We firstly calculate the deformation gradient matrix X0t for each finite element using

$${}_{0}^{t}{\text{X}}_{ij}=\frac{\partial^{t}{\text{x}}_{i}}{\partial^{0}{\text{x}}_{j}}$$ (3)

where 0 and *t* denote the reference and current attributes of nodes respectively, *i* and *j* denote the coordinate dimensional indices.

Then by multiplying a stationary strain-displacement matrix $B0{}_{L0}^{\left(a\right)}$ by X0t, we compute the complete strain-displacement matrix $B0t{}_{L}^{\left(a\right)}$ at any time *t*

$${}_{0}^{t}B_{L}^{\left(a\right)}{=}_{0}B_{L0}^{\left(a\right)}{}_{0}^{t}{\text{X}}^{T}$$ (4)

where *a* ranges from 1 to the nodes’ number per element, $B0{}_{L0}^{\left(a\right)}$ is defined in terms of the spatial derivatives of the element shape functions [22].

The second Piola-Kirchoff stress matrix of the finite element at the integration point is computed as

$$S_{ij}=\mu \left(\delta_{ij}{-}_{0}^{t}C_{ij}^{-1}\right)+\lambda^{t}J{{(}^{t}J-1)}_{0}^{t}\;C_{ij}^{-1}$$ (5)

where **δ**_*ij*_ denotes Kronecker’s delta [22], *λ* and *μ* denote Lame constants.

The nodal reaction forces of each element are computed by using Gaussian quadrature

$${}^{t}F=\int_{0_{V}}{}_{0}^{t}B_{L}^{T}{}_{0}^{t}\hat{S}d^{0}V$$ (6)

where ^0^*V* denotes the element’s initial volume, and $\hat{S}0t$ denotes the vector representation of the second Piola-Kirchoff stress.

Finally, for interfacial and TLED calculation nodes, we compute their net nodal reaction forces ^*t*^**F** at time t by iteratively implementing (Eq 4) for each element. And then we explicitly compute the displacement for each TLED node using central difference formula.

$${}^{t+\Delta t}u_{i}^{\left(k\right)}=\frac{\Delta t^{2}}{M_{k}}\left({}^{t}F_{i}^{external}{-}^{t}F_{i}^{\left(k\right)}\right)+2^{t}u_{i}^{\left(k\right)}{-}^{t-\Delta t}u_{i}^{\left(k\right)}$$ (7)

where *M*_*k*_ denotes the diagonal entry of the *k*th row of the diagonalized mass matrix, $F_{i}^{external}$ denotes the external nodal force.

#### Deformation computation of interfacial and FLSM nodes

The calculation nodes situated in the interface also belong to the regions of FLSM method. Our deformation model computes the deformations of interfacial and FLSM nodes by using the FLSM method. For each region *R*_*i*_ of the FLSM model, we compute the optimal rotation matrix **R**_*r*_ and the translation vectors $C_{r}^{t}$ and $C_{r}^{0}$ by minimizing

$$\sum_{i\in R_{i}}w_{i}\left(R_{r}\left(x_{i}^{0}-C_{r}^{0}\right)+C_{r}^{t}-x_{i}^{t}\right),$$ (8)

where *w*_*i*_ denotes the weight of individual lattice points. The optimal translation vectors are proved to be the mass center vector of the original shape and the mass center vector of the deformed shape, i. e.

$$\begin{array}{ll} C_{r}^{0}=\frac{\sum_{i\in R_{i}}w_{i}x_{i}^{0}}{\sum_{i\in R_{i}}w_{i}}\;, & \;C_{r}^{t}=\frac{\sum_{i\in R_{i}}w_{i}x_{i}^{t}}{\sum_{i\in R_{i}}w_{i}} \end{array}.$$ (9)

For each region, we compute a matrix **A**_*r*_ as

$$A_{r}=\sum_{i\in R_{i}}{\tilde{m}}_{i}\left(x_{i}^{t}-C_{r}^{t}\right){\left(x_{i}^{0}-C_{r}^{0}\right)}^{T}.$$ (10)

We then compute the least squares rotation matrix **R**_*r*_ for all particles of region *R*_*i*_ exploiting the matrix polar decomposition **A**_*r*_ = **R**_*r*_**U**, where **U** denotes a unique third order symmetric stretch matrix. For a region, the least squares rigid transformation of the rest position $x_{i}^{0}$ includes a rotation matrix **R**_*r*_ and a translation that transfers the rotated vector $C_{r}^{0}$ to $C_{r}^{t}$. We use a vector **T**_*r*_ to store this transformation.

$$T_{r}=R_{r}\left(C_{r}^{t}-R_{r}C_{r}^{0}\right).$$ (11)

For each FLSM node or interfacial node, we compute a goal position vector **g**_*i*_ which is the averaged least squares rigid transformation of the original position vector $x_{i}^{0}$ over the regions that the calculation node belongs to.

$$g_{i}=\frac{1}{\left|{ℜ}_{i}\right|}\sum_{r\in {ℜ}_{i}}T_{r}x_{i}^{0}.$$ (12)

According to the FLSM method, for each FLSM node or interfacial node, we update the position vector **x**_*i*_ and the velocity vector **v**_*i*_ based on the goal position vector **g**_*i*_:

$$v_{i}(t+\Delta t)=v_{i}(t)+\partial \frac{g_{i}(t)-x_{i}(t)}{\Delta t}+\frac{F^{external}(t)}{m_{i}}\Delta t,$$ (13)

$$x_{i}(t+\Delta t)=x_{i}(t)+v_{i}(t+\Delta t)\Delta t,$$ (14)

where Δ*t* denotes the time step size.

Compared to our previous work [12, 20], we update the velocity vectors and position vectors of the interfacial calculation nodes by incorporate the reaction forces computed by (Eq 6) into the external forces of (Eq 13), which is a stable position-constrained method rather than a force-based method [12, 20] needing to adjust a user-defined coefficient.

#### Deformation computation of embedded surface

In our hybrid deformation model, both FLSM region and TLED region regularly place the calculation nodes in the form of axis-aligned cubes. The complex surface of object is embedded into the deformable cubes of the hybrid deformation model. For each vertex of the surface, the position vector **x**_*i*_ is calculated as the interpolation of the position vectors of the cube’s nodes.

$$x_{i}=\sum_{j=1}^{8}\varphi_{j}(x_{i})x_{j},$$ (15)

where *φ*(**x**) denotes the weight of each node of the cube.

### Virtual-reality interaction

#### Particle-based interaction simulation

Based on our research on physically based fluid simulation [13, 14], we put forward a new particle-based algorithm for simulating one-way interaction of the reconstructed object and the virtual object, and two-way interaction of particle-based blood and hybrid model-based soft tissue. In our method, motivated by microscopic molecular dynamics, the calculation nodes of hybrid model are considered as Lagrangian particles of fluid, and the adaptively sampled vertices of the reconstructed object surface are considered as solid boundary particles of fluids. Thus, one-way interaction of the reconstructed object and the virtual object can be regarded as one-way fluid-solid interaction, which is directly solved by using multiphase weakly compressible smoothed particle hydrodynamics (WCSPH) [23] solvers.

First, we adopt the method of [24] to adaptively sample particles on the reconstructed object surface. Considering the non-homogeneous distribution of these sampled particles, we adopt the method of [13, 14, 24] to compute the density *ρ*_*i*_ of each particle *i* of the virtual object

$$\rho_{i}=m_{i}\sum_{j}W(x_{ij},h)+\sum_{k}\phi_{b_{k}}(\rho_{0})W(x_{ik},h),$$ (16)

where *x*_*ik*_ = *x*_*i*_−*x*_*k*_, *j* is the index of neighboring particle of the virtual object, *k* is the index of neighboring particle of the reconstructed object, *W* is a Gaussian like kernel function with finite support radius *h*, and $\phi_{b_{k}}(\rho_{0})=\rho_{0}V_{b_{k}}$ scales the contribution of particle *k* to particle *i*. $V_{b_{k}}$ is the current volume value of particle *k*.

Then, we compute the interaction force $F_{i\leftarrow k}^{c}$ exerted by particle *k* of the reconstructed object to particle *i* of the virtual object. The interaction force $F_{i\leftarrow k}^{c}$ includes the normal force $F_{i\leftarrow k}^{p}$ and the tangential force $F_{i\leftarrow k}^{v}$, which can be computed by solving Navier-Stokes equations using WCSPH method

$$F_{i\leftarrow k}^{\text{p}}=-m_{i}\phi_{b_{k}}(\rho_{0})\frac{p_{i}}{\rho_{i}^{2}}\frac{x_{ik}}{\left|x_{ik}\right|}W(x_{ik},h),$$ (17)

$$F_{i\leftarrow k}^{\text{v}}=\mu m_{i}\phi_{b_{k}}(\rho_{0})\frac{v_{ki}}{\rho_{i}^{2}}\nabla^{2}W(x_{ik},h),$$ (18)

where *μ* is surface friction coefficient, *p* is fluid pressure which is computed by Tait’s equation of [23], and *v* is velocity. In our framework, for a particle *k* of the reconstructed object, its velocity *v*_*k*_ is computed as

$$v_{k}=\frac{x^{cur}-x^{pre}}{\Delta t},$$ (19)

where *x*^*cur*^ is the centre of gravity of the current reconstructed object, and *x*^*pre*^ is the recorded centre of gravity of the reconstructed object in the previous frame.

However, when the velocity difference becomes relatively larger, only relying on the above interaction forces results in the virtual object penetrating into the surface of the reconstructed object. To solve these problems, we incorporate the velocity-position correction scheme of [13] and [14] into our multi-GPU virtual-reality interaction framework.

**Fig 6:**
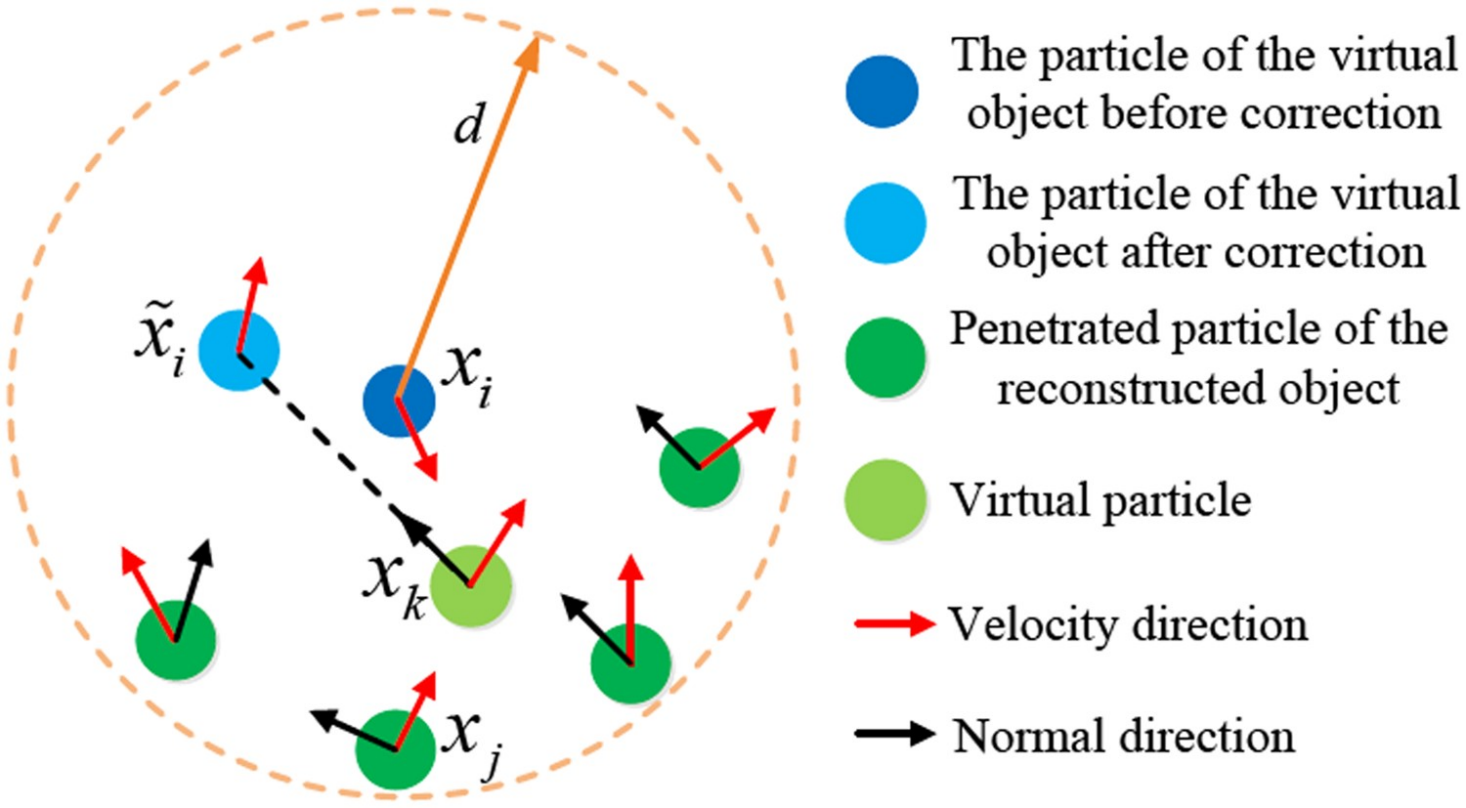
Velocity-position correction for avoiding penetrations.

For a particle *i* of the virtual object and a particle *j* of the reconstructed object, if there exists *v*_*ij*_ ⋅ *n*_*j*_ < 0 and |*x*_*ij*_| < *d*, the reconstructed object is considered to be penetrated by the virtual object at the position *x*_*j*_. *d* is a user-defined value, and *n*_*j*_ denotes the surface normal of the reconstructed object at *x*_*j*_. Fig 6 shows that a particle *i* could penetrate more than one particle of the reconstructed object at the same time. In this case, we dynamically create a virtual boundary particle *k* for the reconstructed object to collide with particle *i*. For this virtual particle *k*, its filed quantity *A*_*k*_, such as normal, position and velocity, is computed as the weighted average of the corresponding values of the neighboring penetrated particles:

$$A_{k}=\frac{\sum_{j}m_{j}A_{j}W(x_{ij},d)}{\sum_{j}m_{j}W(x_{ij},d)}.$$ (20)

After the time integration of the deformation simulation, we first correct the position of *i* along the normal of *k*

$${\tilde{x}}_{i}=x_{k}+dn_{k},$$ (21)

where ${\tilde{x}}_{i}$ is the corrected position of *i*.

We then correct the velocity of *i* based on boundary material property and momentum conservation law. The velocities of *i* and *k* are projected onto both the normal direction and the tangential direction of *k*, so we get four velocity components: $v_{i}^{n}$, $v_{i}^{t}$, $v_{k}^{n}$ and $v_{k}^{t}$. By enforcing momentum conservation constraint along these two directions, we obtain the following formulas:

$$m_{i}v_{i}^{n}+m_{k}v_{k}^{n}=m_{i}{\tilde{v}}_{i}^{n}+m_{k}{\tilde{v}}_{k}^{n},$$ (22)

$$m_{i}v_{i}^{t}+m_{k}v_{k}^{t}=m_{i}{\tilde{v}}_{i}^{t}+m_{k}{\tilde{v}}_{k}^{t},$$ (23)

where ${\tilde{v}}_{i}^{n}$, ${\tilde{v}}_{k}^{n}$, ${\tilde{v}}_{i}^{t}$ and ${\tilde{v}}_{k}^{t}$ are corrected velocities.

Because the position and velocity of the reconstructed object only depend on the real object, the virtual-reality coupling is one-way. Thus we set the corrected velocities of particle *k*: ${\tilde{v}}_{k}^{n}={\left(0,0,0\right)}^{T}$ and ${\tilde{v}}_{k}^{t}={\left(0,0,0\right)}^{T}$.

Substituting it into Eqs (22) and (23), we get

$${\tilde{v}}_{i}^{n}=\frac{m_{i}v_{i}^{n}+m_{k}v_{k}^{n}}{m_{i}},$$ (24)

$${\tilde{v}}_{i}^{t}=\frac{m_{i}v_{i}^{t}+m_{k}v_{k}^{t}}{m_{i}}.$$ (25)

As for two-way coupling of particle-based blood and hybrid model-based soft tissue, we also use the above coupling method. The differences are the nodes of hybrid model-based soft tissue have their own velocities, and the corrected velocities for the virtual particle are set according to the method of [13].

#### Virtual cutting simulation

**Fig 7:**
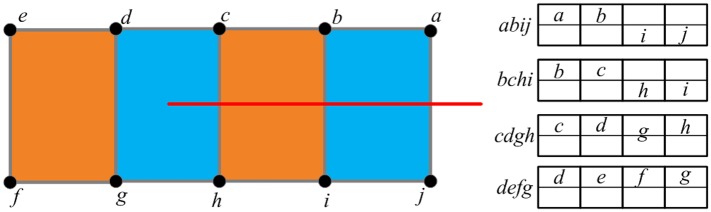
Finding the completely cut elements.

**Fig 8:**
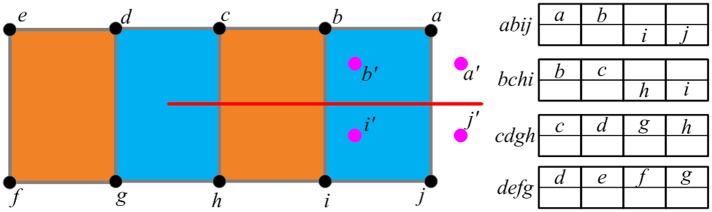
Generating the virtual nodes for the nodes of completely cut elements.

**Fig 9:**
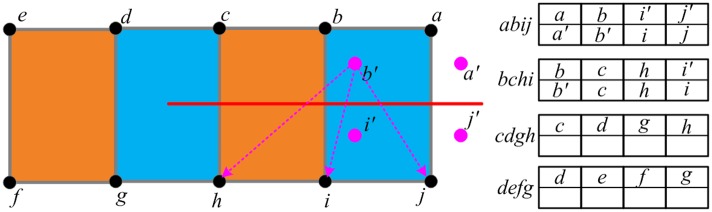
Allocating the virtual nodes to generate new elements.

**Fig 10:**
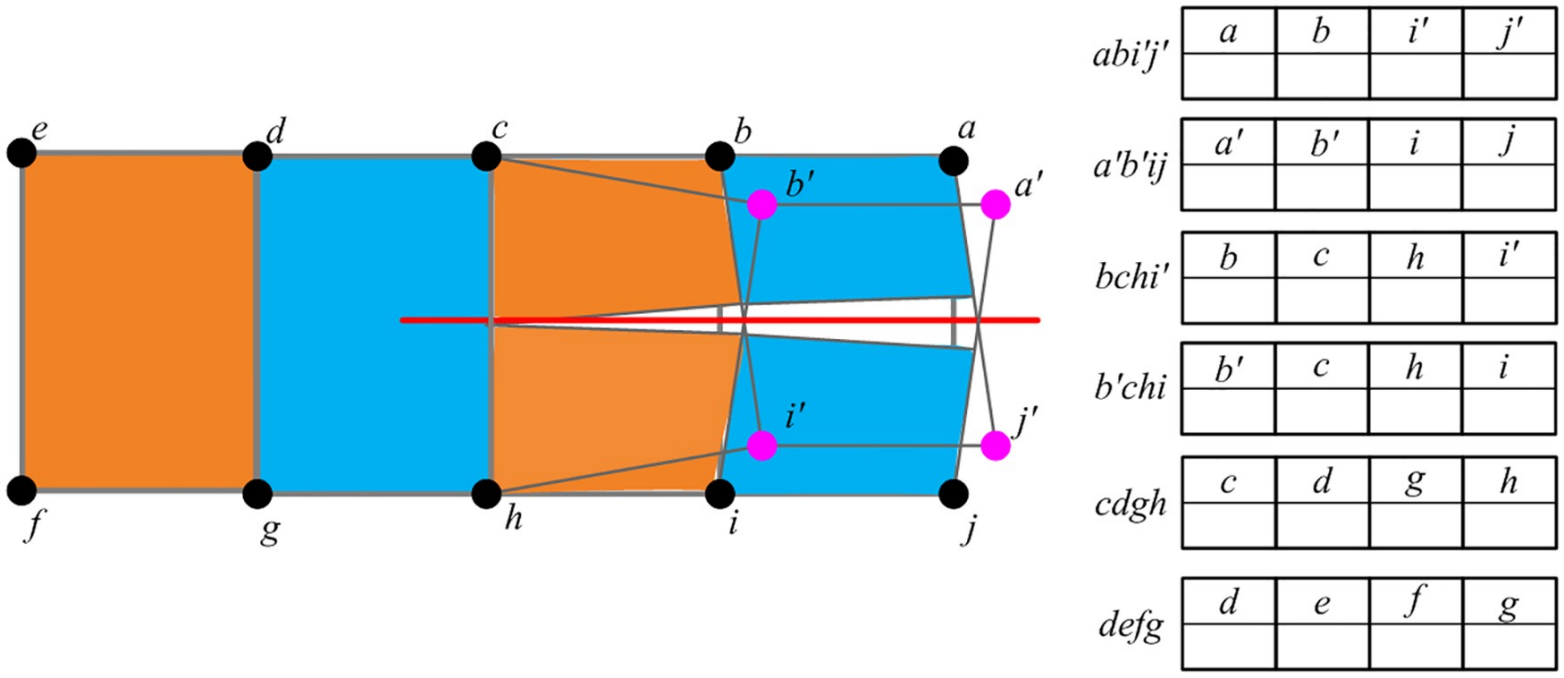
Creating new generated elements.

Virtual cutting of deformable objects is one of the important 3D interactive manipulations in virtual-reality interaction environments. Based on our previous work [12, 20], by combining the improved hybrid deformation model and the virtual node algorithm [25], we propose to use the scalpel reconstructed from real images to cut the virtual deformable objects. In our virtual-reality interaction framework, the cut operation of the reconstructed scalpel is restricted to the region simulated by TLED method. The cutting procedure includes 4 steps:

Step 1. Finding the elements which are completely cut
  Firstly, it implements the collision detection procedure to find the elements which intersect with the cutting plane swept by the reconstructed scalpel. Spatial hashing algorithm is adopted to accelerate time-consuming collision detection. As shown in Fig 7, after collision detection, we find that three elements intersect with the cutting plane: *abij*, *bchi*, and *cdgh*. Among these three elements, our method regards *abij* and *bchi* as the completely cut elements, because the graph structures formed by their 4 nodes are unconnected. For the completely cut elements, our method allocates two arrays to separately store the information of nodes located in each side of the cutting plane.
Step 2. Generating the virtual nodes
  Fig 8 shows the principle of generating virtual nodes for the completely cut elements. For each node of the completely cut elements, if all its attached elements are completely cut elements, our method generates a virtual node for it. As shown in Fig 7, for *a*, *b*, *i* and *j*, our method generates *a*′, *b*′, *i*′ and *j*′ as their virtual node respectively.
Step 3. Allocating the virtual nodes
  When the virtual nodes are generated, our method allocates these nodes in the arrays of the completely cut elements. As shown in Fig 9, for the completely cut element *abij*, the node *a* stored in the first array has a virtual node *a*′, so we fill the virtual node *a*′ into the corresponding position of the second array. As for the node *c*, because it does not has the virtual node, we fill it into the second array of the element *bchi*.
Step 4. Creating new generated elements
  As shown in Fig 10, for each array of the completely cut elements, our method creates a new element according to the node index. Then the pre-computed quantities are copied from the completely cut element to the newly generated elements. Each new element contains a part of material of the completely cut element, such as mass. For each new element, by only rendering the part situated at the side of the cutting plane, our method produces very smooth surface of the incision.

### Multi-GPU implementation

**Fig 11:**
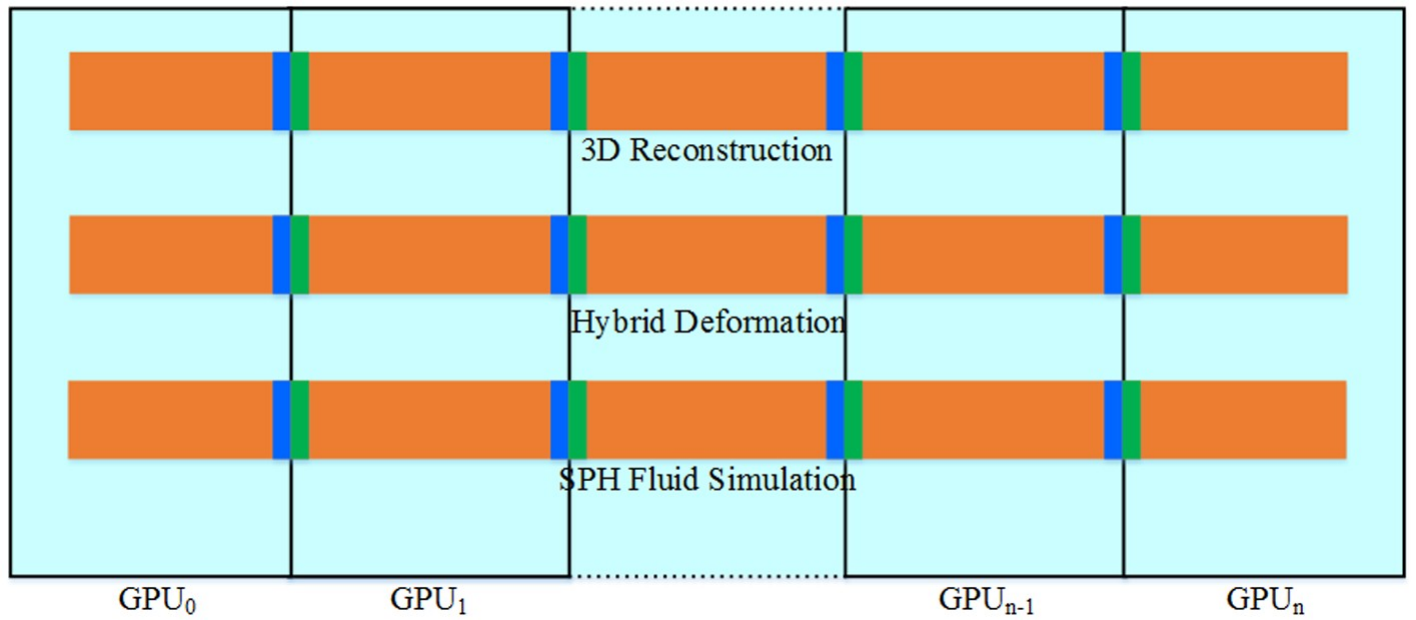
Multi-GPU implementation method.

The proposed virtual-reality interaction simulation framework is entirely implemented on the multi-GPU platform by using CUDA for the real-time performance. As the memory between GPUs is not shared, parallelism across multiple GPUs would be less fine grained than that on a single GPU to reduce the amount of interaction and number of memory copies between each GPU. As shown in Fig 11, our multi-GPU implementation method respectively splits the workload of 3D reconstruction, hybrid deformation and fluid simulation into many smaller parts that can be computed respectively on different GPUs. The number of subtasks equals the number of GPUs. The halo of the subspace is represented by the colored edges in Fig 10. Each subspace holds a copy of the edge of its adjacent subspaces to take fast neighbor access into account. Because the neighbor information of each computation node is not changed during the simulation, the computation spaces of 3D reconstruction and hybrid deformation are evenly split into subspaces and then distributed to each GPU. As for SPH fluid simulation, the particles freely moving in space require the solver to frequently update the corresponding workload of the respective GPUs. In this paper, we adopt the sophisticated load balancing strategy of [26] to achieve optimal performance, which frequently implements a domain decomposition correction to adjust the current distribution of the workloads among the GPUs.

Multi-GPU implementation using CUDA is especially suitable for addressing the problems that can be indicated as data-parallel computations, so during pre-processing phase, the data elements should be made parallel and independent. The reconstruction space is divided into uniform and independent voxels with certain resolution. Then projections of each voxel onto every image plane are calculated according to the calibration information. Because the data access is stochastic during execution, the data should be stored into texture memory of GPU rather than global memory which gets maximum memory bandwidth only when following coalesced access pattern. Other information should also be stored into texture memory, such as look-up tables of marching cube algorithm, binary image of received foreground silhouette, and pre-computed data for hybrid deformation model.

**Fig 12:**
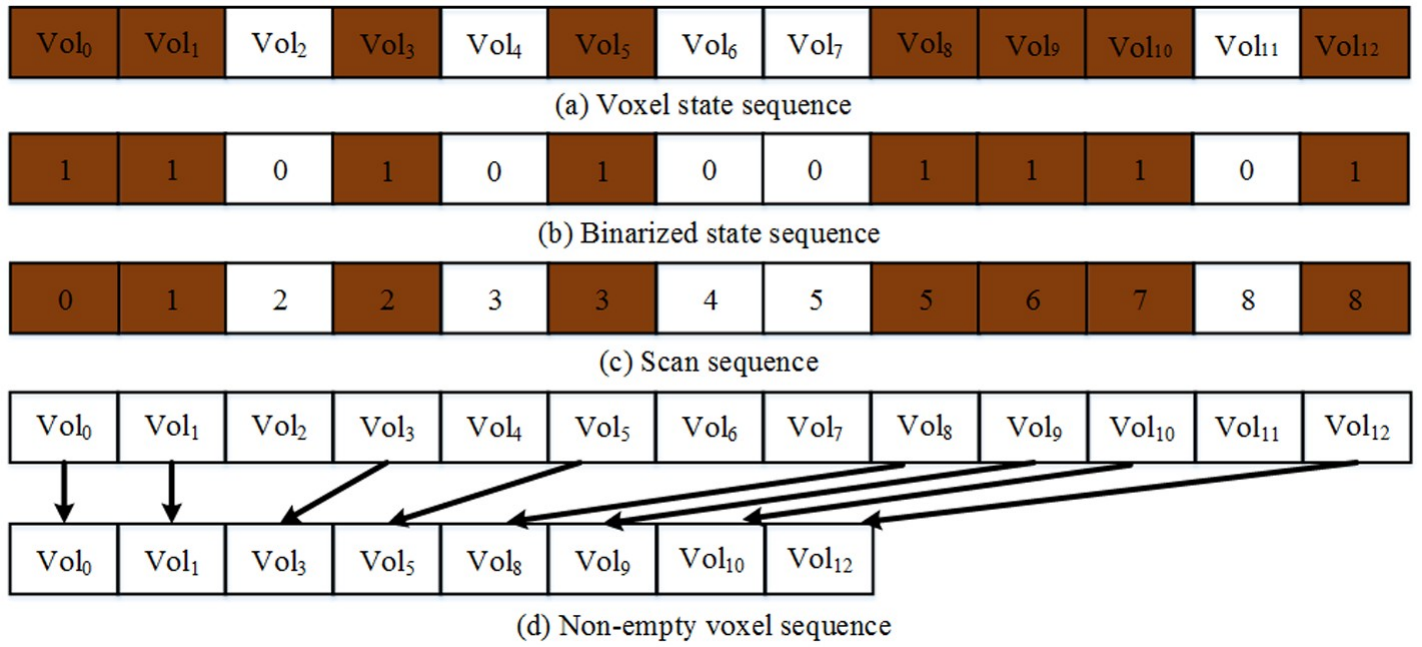
Parallel processing of voxel state sequence.

The intersection test of voxels’ projections and foreground silhouettes is an important content for visual hull computation. We define a kernel named *classifyVoxels()* executed for each voxel, and obtain a voxel state sequence shown in Fig 12(a). The voxels marked as ***Inside*** and ***Outside*** have no intersections with the reconstructed objects’ surface, so voxels of these two types are labeled as 0, the ***Uncertain*** voxels having intersections with the objects’ surface are labeled as 1. So as shown in Fig 12(b), the voxels’ state sequence is binarized parallelly, thus it is composed of digits 0 and 1. The triangularized isosurface should be extracted from the ***Uncertain*** voxels which are set to 1 in the binarized state sequence, but in fact most of the voxels are set to 0. Thus, to improve the performance, we must compress the voxels’ status sequence and remove the digits 0. Our method uses the *cudppScan()* function of CUDPP library to implement parallel prefix sum on GPU and get the scan sequence of the voxels’ status sequence (Fig 12(c)). Then we launch *compactVoxels()* kernel to implement parallel reduction, and gets the ***Uncertain*** voxel sequence (Fig 12(d)).

Based on NVIDIA CUDA ‘Marching Cubes Isosurfaces’ demo, the exact intersection points on the edges of the ***Uncertain*** voxels are computed, and then a triangular mesh surface is extracted by connecting these intersection points. The computations are implemented in parallel on GPU by launching the *generateTriangles()* kernel. During parallel triangulation, the buffer object VBO is registered and mapped by using the mutual-operation property of CUDA and OpenGL. When the position vector, normal vector and texture coordinate of each triangular vertex are computed by using CUDA, these data are transferred to OpenGL through VBO.

Efficient texture mapping improves the visual quality of the reconstructed objects. In image-based modeling system, the textures derive from the foregrounds of images captured by different cameras, so each triangle has many possible mapped textures. We must store all textures and choose the most suitable one. Based on *Multitexture Mapping* and programmability of current GPU, we present a new texture mapping method applied to image-based modeling system. Firstly, the method enables the *Multitexture Mapping* and sends the normals and texture coordinates of each triangular vertices stored in buffer objects. Then the vertex shader programmed by cg language outputs the normals and texture coordinates. The pixel shader chooses the suitable texture of each fragment according to the angles between the normal and every optical axis of the cameras. This shader reads the texture from texture sampler variables which must be provided by the application as uniform parameters to a Cg program.

**Fig 13:**
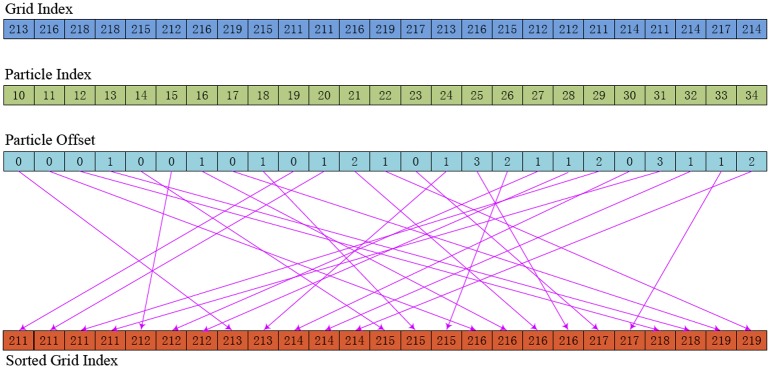
Neighboring particle search method.

To simulate virtual-reality interaction, the search of neighboring particles for each particle is required in each time step, which is the most time-consuming component. In order to accelerate this computation, based on the uniform spatial hashing algorithm using *Z-indexing* [27], we propose a new GPU-based neighboring particle search method. We find that the order of the particles in a grid cell does not affect neighboring particle search. As shown in Fig 13, different from the method of [27], our method only implements once *Z-indexing* sort operation for grid cells, but not for particles to avoid additional sorting: First, we parallelly compute the number of particles in each grid cell. Then, our method implements parallel prefix sum on GPU and sorts for particle array. For example, the grid cell with an index of 211 has 4 particles, the grid cell with an index of 212 has 3 particles, and so on. Finally, we implement radix sorting for grid cells based on Z-indexing. Each particle has an offset value relative to the starting position in the sorted grid index array, and thus we can parallelly insert the particle into the sorted array avoiding access conflicts.

## Results and analysis

We have tested the multi-GPU accelerated virtual-reality interaction simulation method via several example scenarios. The hardware and software configuration of our multi-GPU accelerated virtual-reality interaction framework is shown in Table 1.

**Table 1:** Experimental environment.

Item	Description	Number
Client	Intel Core 2 Duo E6400 2.13 GHz, 4GB DDR2-667 memory, WIN7 PRO 64. IDE: Visual Studio 2013, OpenCV 2.0.	8
Server	Intel Core 2 Duo E6400 3.5 GHz, 16GB DDR2-667 memory, WIN7 PRO 64, 6x NVIDIA Geforce GTX 650. IDE: Visual Studio 2013, OpenGL 4.0, CUDA 7.0, Cg 3.0.	1
Optical Camera	PointGrey Flee2, resolution 640x480, 30fps	8
TOF Depth Camera	PMD CamCube 2.0, resolution 200x200, 30fps	1
Network	1Gbit Ethernet	/

### Reconstruction and interaction results

**Fig 14:**
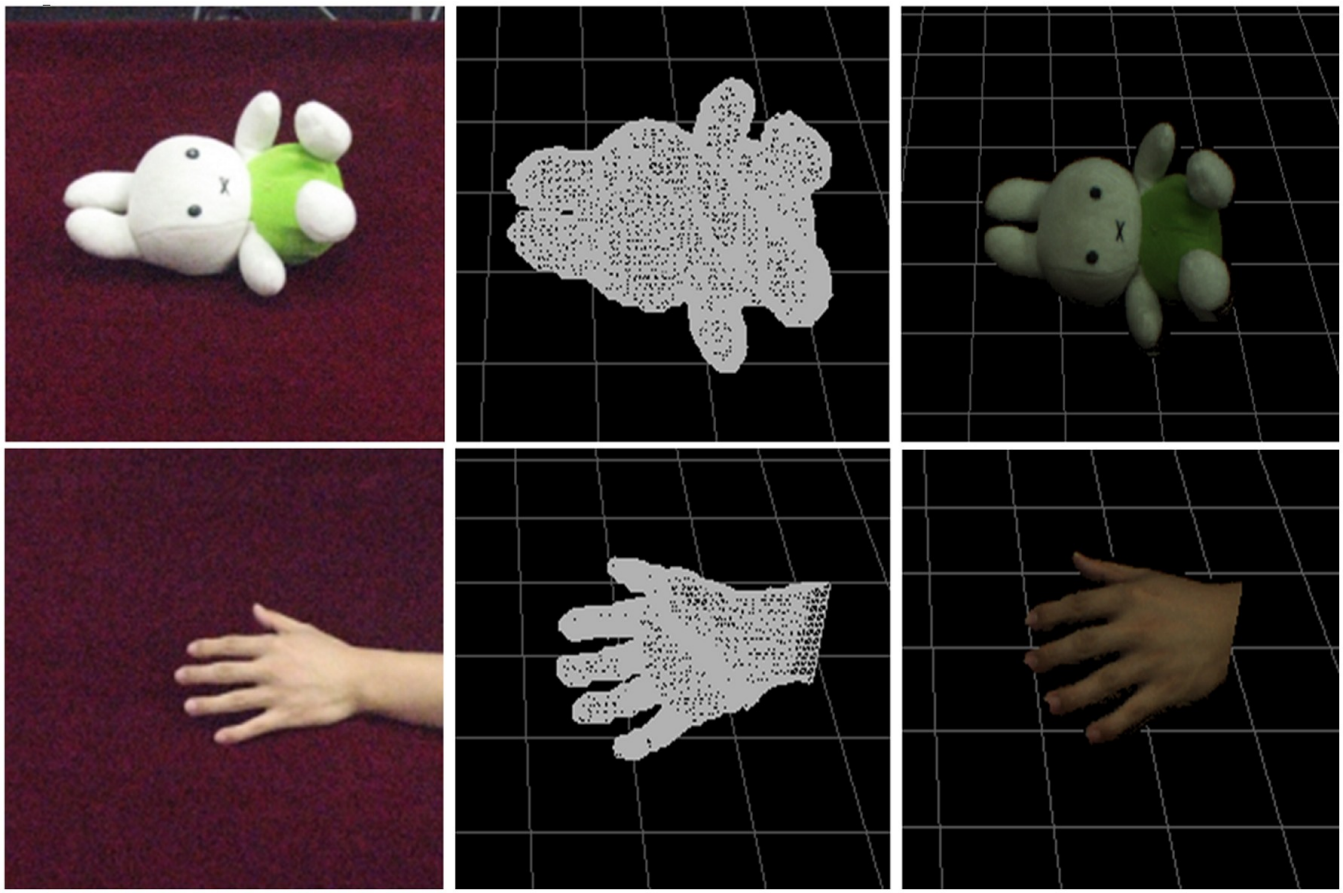
The reconstructed results of bunny and hand.

Fig 14 shows the reconstructed results of two real objects placed in the 1m×1m×1m sized frame: a toy bunny (Top) and a real hand (Bottom). From left to right: the captured image, the triangle mesh surface of the reconstructed object and the reconstructed object surface with texture mapping. In this example, the input image resolution is 640*480, and the division resolution of the real space is 64*64*64. The frame rate can be achieved 35.0 *FPS*.

**Fig 15:**
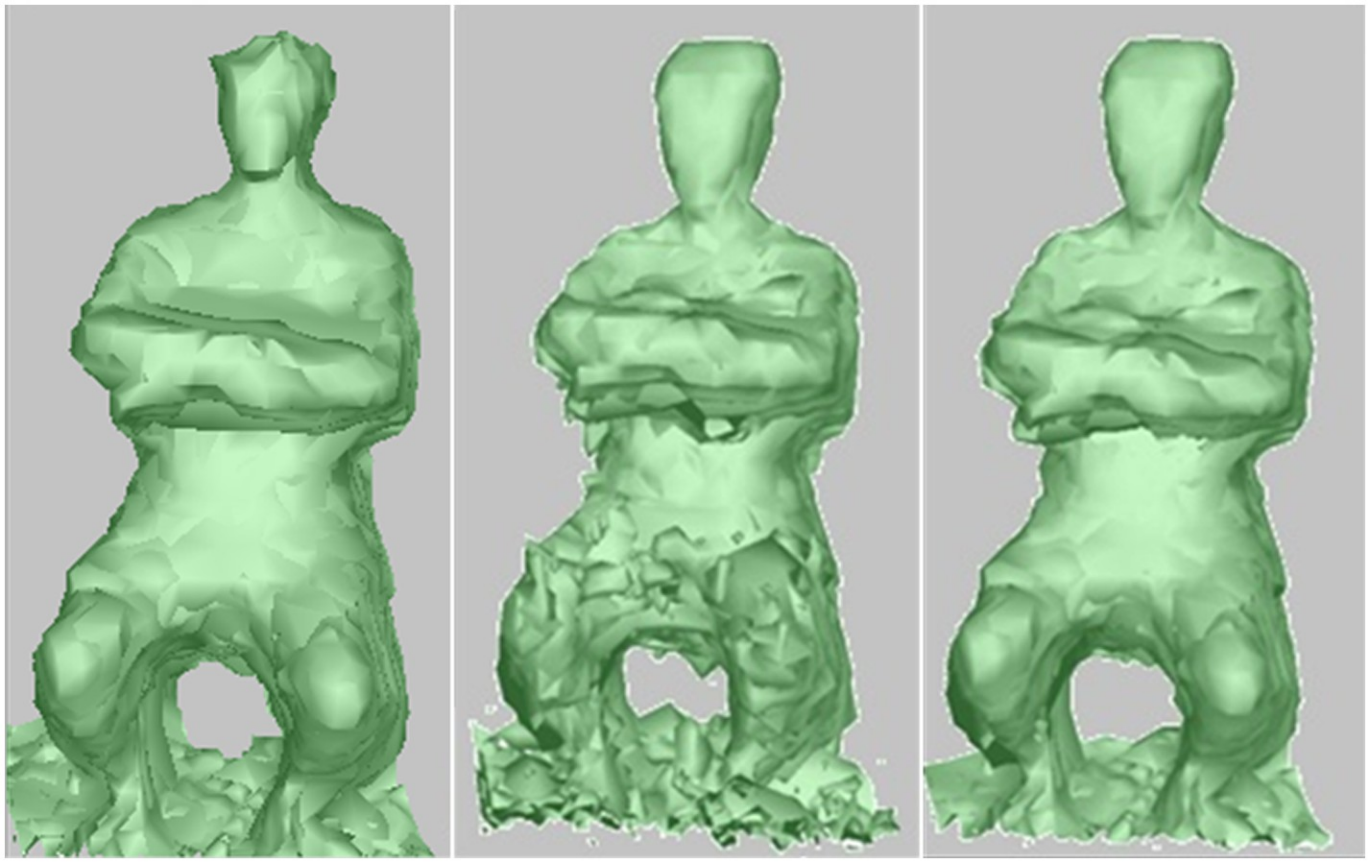
Comparing the reconstruction result with traditional VBVH method.

Fig 15 shows the comparison results of the proposed reconstruction method and the previous VBVH reconstruction methods. From left to right: the reconstruction result of Kim’s method [28], the reconstruction result of Shao’s method[14], and the reconstruction result of our VBVH method by using our 3*m*×3*m*×3*m* sized frame. Kim’s method produces smooth surface by using multiple view stereo matching algorithm, but many vertices are mistakenly deleted. Shao’s method generates many reconstructed noises which reduces the smoothness of the surface. In contrast, by exploiting the data of TOF depth camera to correct the triangulated surface generated by MC algorithm, our extended VbVH method can produce more smooth and accurate reconstruction results. Compared to Zuo’s method [29], our reconstruction method can not handle concave and non-Lambertian objects, but can meet real-time performance requirements by using multiple GPUs.

**Fig 16:**
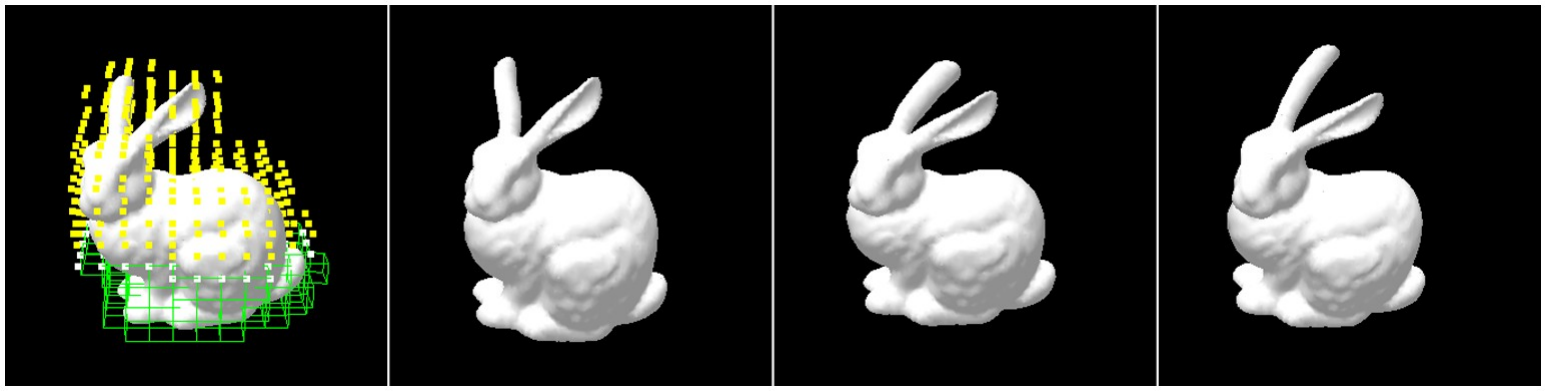
The deformations of bunny modeled by the improved hybrid method.

Fig 16 shows the deformation results of the bunny modeled by our improved hybrid deformation method. The ratio of TLED nodes and LSM nodes is 6:4. From the animation results, our method could smoothly model the larger deformations of the object divided into two types of regions.

**Fig 17:**
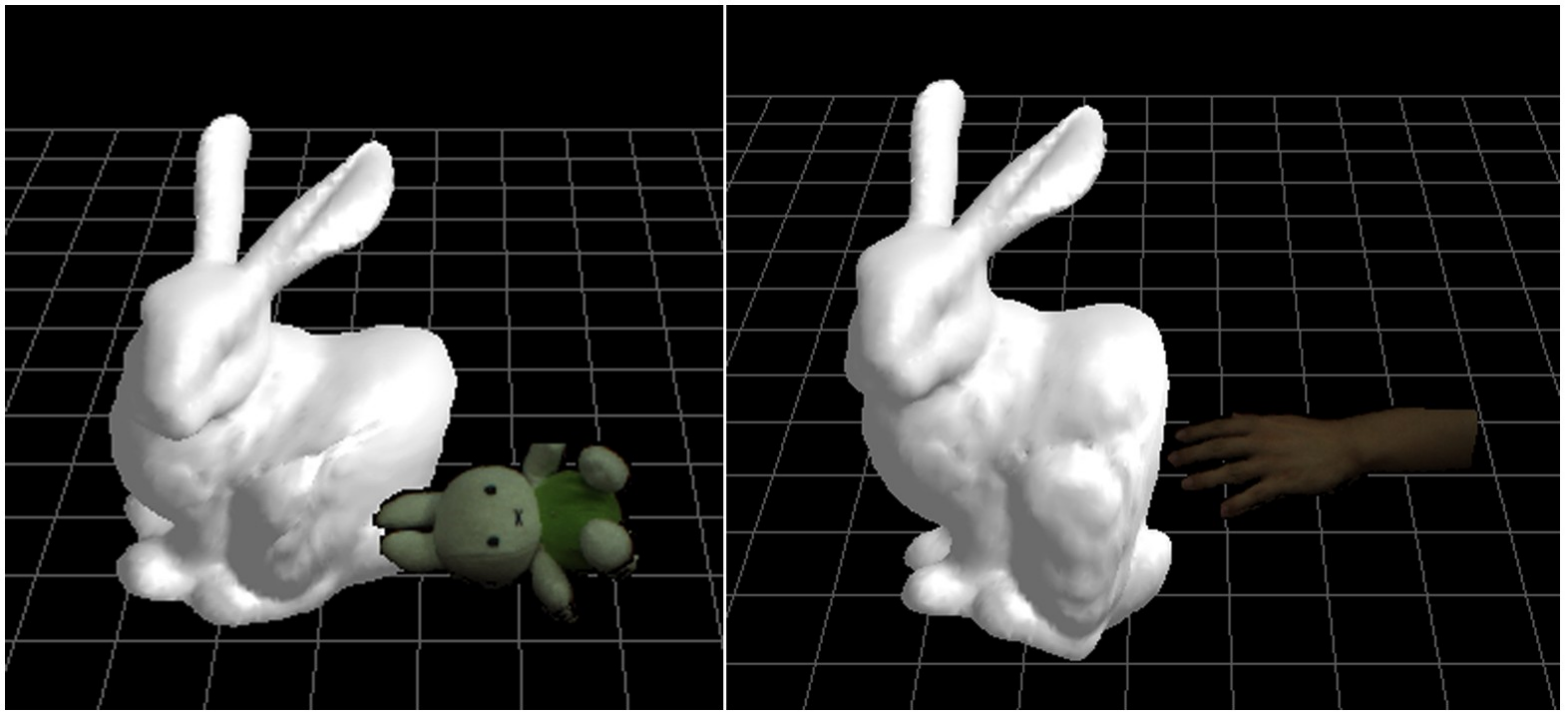
The reconstructed objects interact with the virtual deformable bunny.

Fig 17 shows two virtual-reality interaction examples for the 1*m*×1*m*×1*m* sized frame. For the first example, we let the reconstructed toy bunny to collide with the virtual bunny animated by the improved hybrid model. The second example shows the reconstructed hand touches the virtual bunny. The results demonstrate that our method can effectively simulate virtual-reality interaction. Especially, the penetration artefacts are avoided in the interaction simulation.

**Fig 18:**
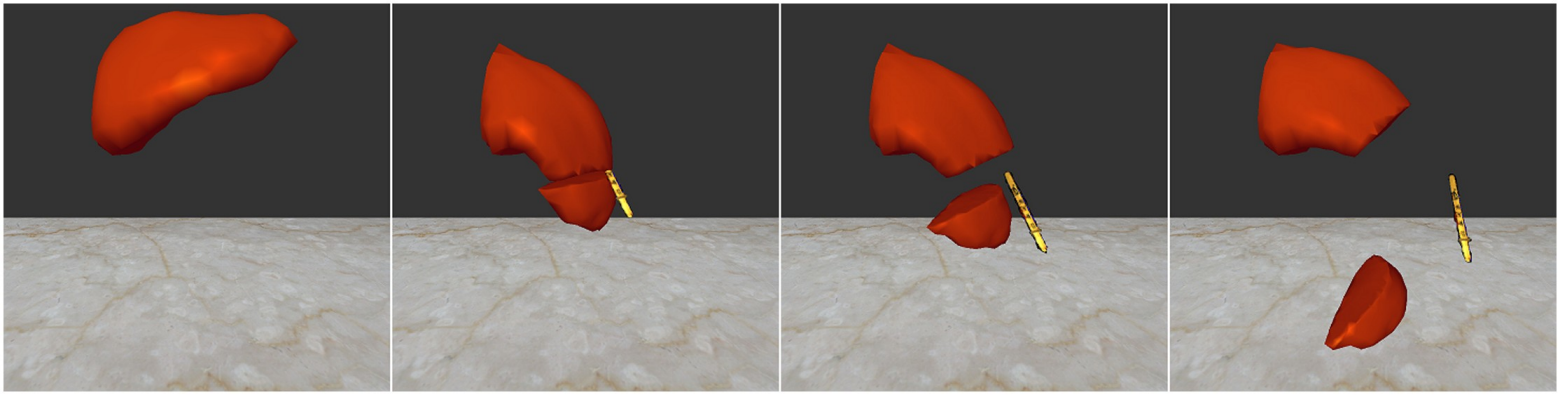
Cutting the virtual liver using the reconstructed scalpel.

Fig 18 is a scenario that the reconstructed scalpel cuts the virtual liver fixed at one end. From the results, our improved hybrid deformation method could stably model the larger deformations of virtual soft tissues, and our cutting method could create smooth cutting incisions. The division resolution of the real space is 64*64*64, and the ratio of TLED nodes and LSM nodes is 7:3. In the simulation of virtual cutting, no texture mapping is used for the surface of the virtual object.

**Fig 19:**
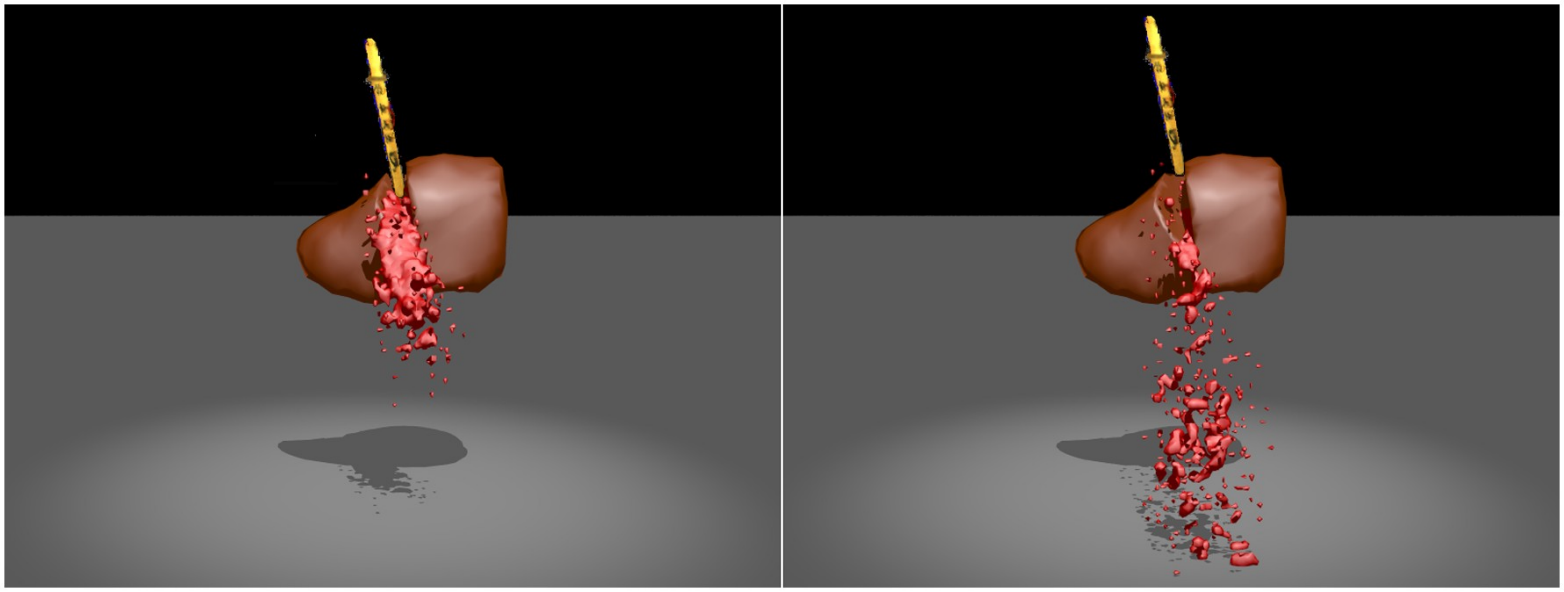
The virtual cutting simulation with bleeding effects.

Fig 19 shows that the reconstructed scalpel cuts the virtual liver with the simulation of bleed effects. From the results, we can conclude that our virtual cutting method can produce smooth incisions, and our virtual-reality interaction method can realistically and stably simulate one-way coupling of the reconstructed object and the virtual object, and two-way coupling of particle-based blood and hybrid model-based soft tissue. The division resolution of the real space is 64*64*64, and the maximum number of SPH particles is 40*k*. Compared to the virtual cutting simulator using the haptic feedback devices [30], the obvious limitation of our framework is that the cutting plane cannot be accurately calculated from the reconstructed scalpel, but the virtual-reality interaction model can be applied to the remote virtual surgery.

### Time performance analysis

The division resolution of the real space is a critical influence factor of the time performance of the VbVH based reconstruction process. Table 2 shows the comparisons of time performance between CPU and multi-GPU implementation of the reconstruction process in different division resolutions. From the results, our multi-GPU based VbVH reconstruction method can obtain real-time performance for moderately complicated computations. Compared to the CPU implementation method, our multi-GPU implementation method achieves impressive speedups of about 14 times for the division resolution 100*100*100. And compared to the single-GPU implementation method, our multi-GPU implementation method achieves speedups of about 3.5 times.

**Table 2:** Time performance comparison of 3D reconstruction methods(ms).

3D reconstruction	64*64*64		100*100*100	
	Hand	Bunny	Hand	Bunny
CPU version	180.4	194.6	554.5	596.8
Single-GPU version	57.6	64.4	137.5	149.8
Multi-GPU version	26.2	27.5	39.3	41.5

**Fig 20:**
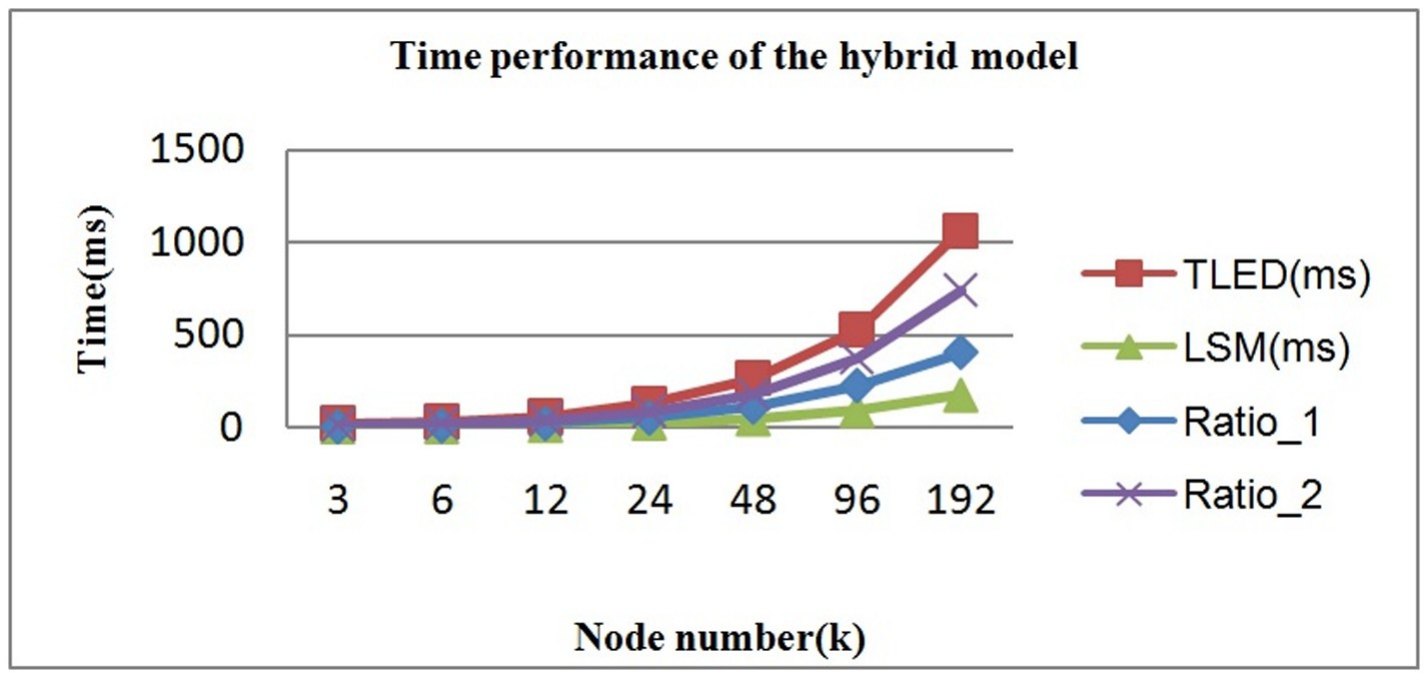
Time performance of the improved hybrid deformation model.

For the deformation simulation, our improved hybrid method could simultaneously maintain the physical accuracy of TLED and the computational efficiency of FLSM. We could regulate the balance between computational efficiency and physical accuracy through dynamically adjusting the ratio of FLSM nodes and TLED nodes. Fig 20 shows the time performance of the hybrid deformation model using different ratios of TLED nodes and FLSM nodes. Ratio_1 is set to 7:3, and Ratio_2 is set to 3:7.

Our multi-GPU implementation method can significantly improve the time performance of each part of the proposed virtual-reality interaction framework. Table 3 shows that the multi-GPU implementation of our texture mapping method is much faster than the CPU implementation and the single-GPU implementation. The maximum acceleration ratios are 7.5 and 1.8 respectively for the division resolution 64*64*64. From the results shown in Table 4, compared to the GPU implementation of Z-index sorting method [27] and uniform space partition method [13], our new multi-GPU based method significantly improves the time performance of neighboring particle search.

**Table 3:** Time performance comparison of texture mapping method(ms).

Texture mapping	64*64*64		100*100*100	
	Hand	Bunny	Hand	Bunny
CPU version	52.5	56.7	207.4	221.3
Single-GPU version	13.2	14.7	46.9	50.6
Multi-GPU version	9.4	9.8	27.6	28.1

**Table 4:** Time performance comparison of neighboring particle search methods(ms).

Particle number	Goswami’s method [27]	Shao’s method[13]	Our method
300 *k*	2.9	3.8	2.4
400 *k*	4.2	5.3	3.5
500 *k*	5.5	6.4	4.2
600 *k*	6.4	7.2	4.7

In Table 5, we demonstrate the time performance comparison of CPU based and multi-GPU based virtual-reality interaction framework. From the results, our multi-GPU implementation method of the whole virtual-reality interaction framework significantly improves the time performance. The maximum acceleration ratio is 14.5. And compared to the single-GPU based virtual-reality interaction framework of [14], our method also has obvious performance advantages. Under the moderate calculation scale, the frame rate of our method can be achieved about 25 *FPS*, which is suitable for real-time virtual reality applications.

**Table 5:** Time performance comparison of virtual-reality interactions(ms).

Virtual-reality interaction	64*64*64			100*100*100		
	Fig 17	Fig 18	Fig 19	Fig 17	Fig 18	Fig 19
CPU version	275.6	350.4	392.5	995.0	1278.6	1563.5
Multi-GPU version	37.4	43.2	45.6	78.5	92.3	103.8
Single-GPU method [14]	56.3	65.4	67.8	96.5	111.7	125.6

## Conclusion

We have designed a real-time multi-GPU accelerated virtual-reality interaction simulation framework where the reconstructed objects from camera images interact with virtual deformable objects. It glues image-based 3D reconstruction, hybrid model based deformation, virtual cutting, particle-based coupling and parallel computation on multiple GPUs for a new immersive interactive experience. The experiment results have demonstrated that our multi-GPU accelerated virtual-reality interaction framework achieves real-time performance under the moderate calculation scale, which provides a new effective 3D interaction technique for virtual reality applications.

In future, our efforts are geared towards simulating more types of virtual-reality interactions and determining more sophisticated load balancing strategy for multi-GPU architecture.

## Supporting information

S1 File 3D reconstruction experiment code. (ZIP)

S2 File Virtual cutting experiment code. (ZIP)
