# Supplementary figures and images for: A multi-GPU accelerated virtual-reality interaction simulation framework
Source: PLoS One. 2019 Apr 11;14(4):e0214852. doi: 10.1371/journal.pone.0214852 (PMC6459549; doi:10.1371/journal.pone.0214852)

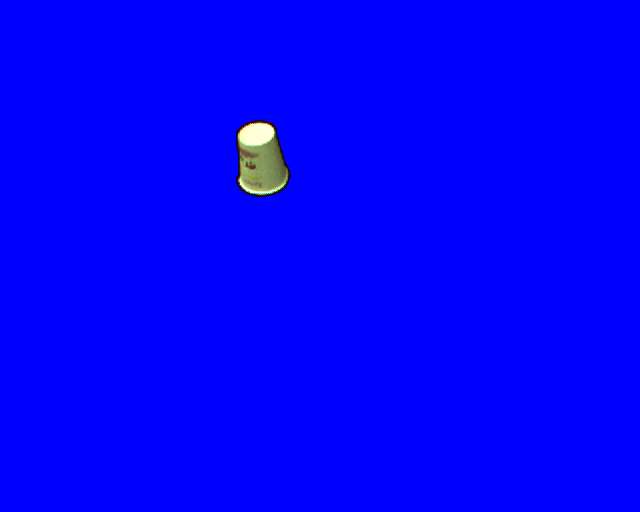

Supplement: S1 File — (ZIP) [file pone.0214852.s001.zip › 3D Reconstruction Experiment code/data/1_1.bmp]

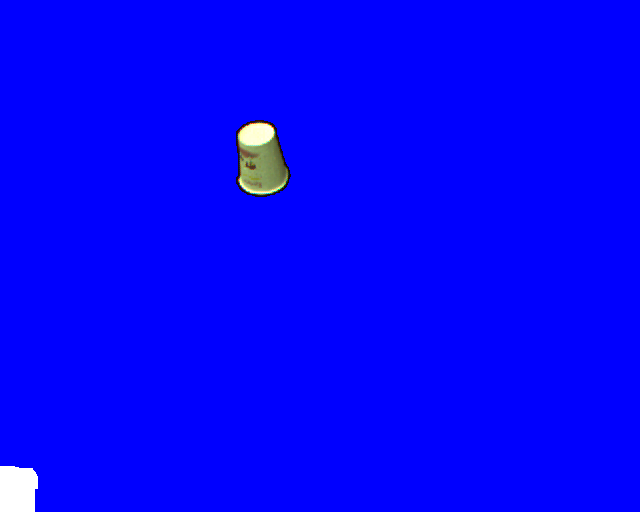

Supplement: S1 File — (ZIP) [file pone.0214852.s001.zip › 3D Reconstruction Experiment code/data/1_11.bmp]

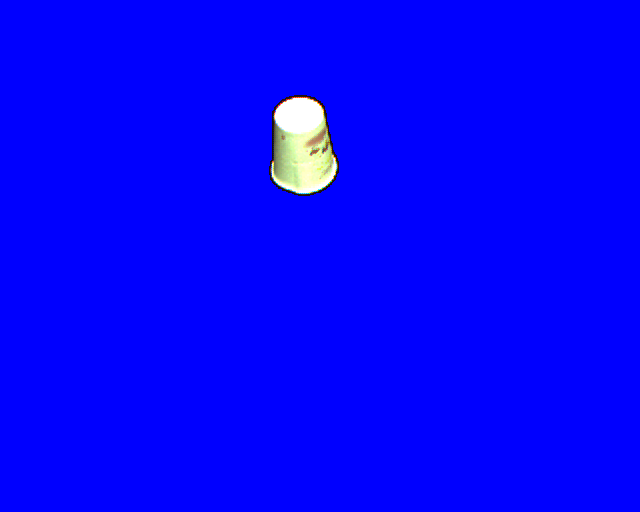

Supplement: S1 File — (ZIP) [file pone.0214852.s001.zip › 3D Reconstruction Experiment code/data/1_2.bmp]

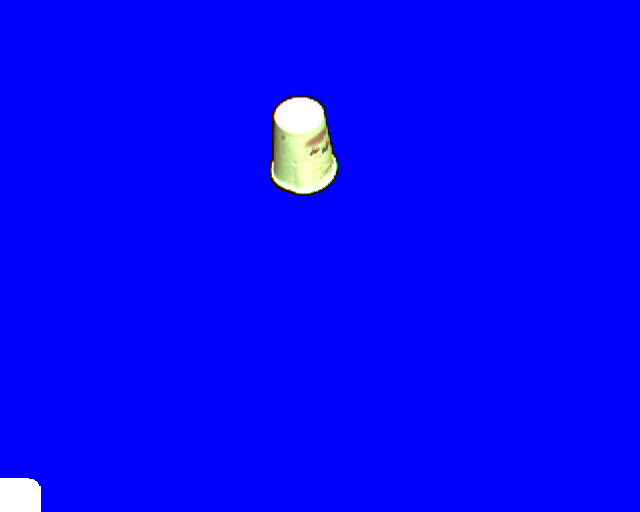

Supplement: S1 File — (ZIP) [file pone.0214852.s001.zip › 3D Reconstruction Experiment code/data/1_22.bmp]

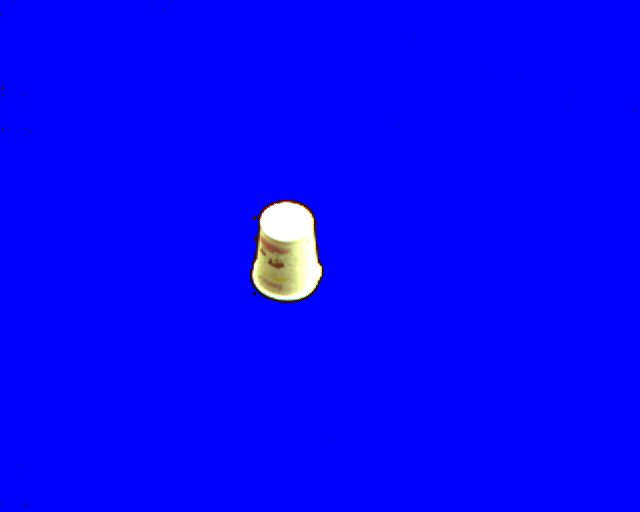

Supplement: S1 File — (ZIP) [file pone.0214852.s001.zip › 3D Reconstruction Experiment code/data/1_3.bmp]

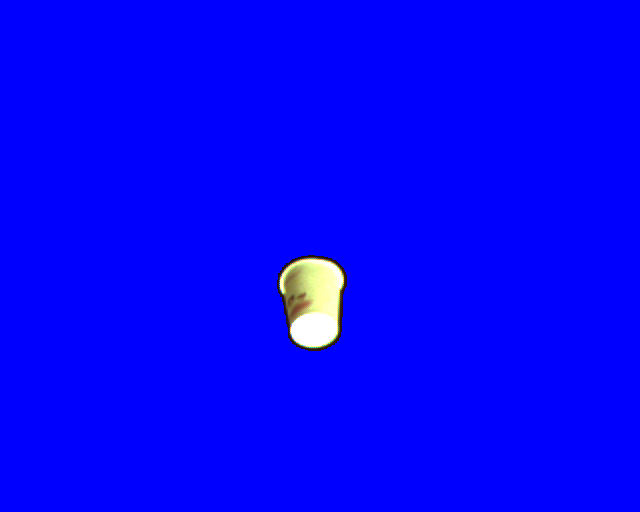

Supplement: S1 File — (ZIP) [file pone.0214852.s001.zip › 3D Reconstruction Experiment code/data/1_4.bmp]

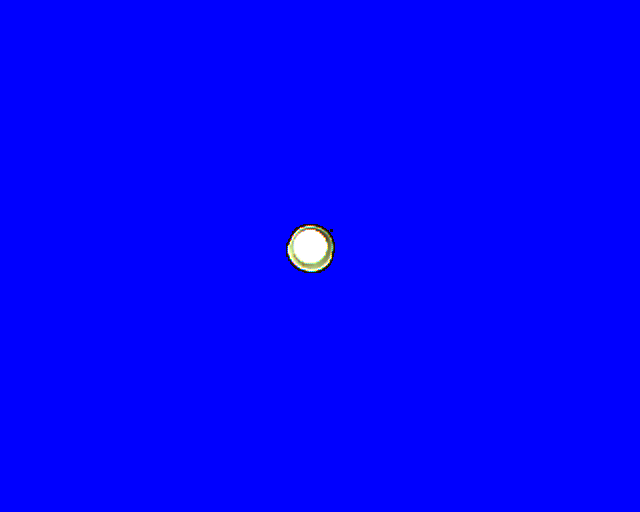

Supplement: S1 File — (ZIP) [file pone.0214852.s001.zip › 3D Reconstruction Experiment code/data/1_5.bmp]

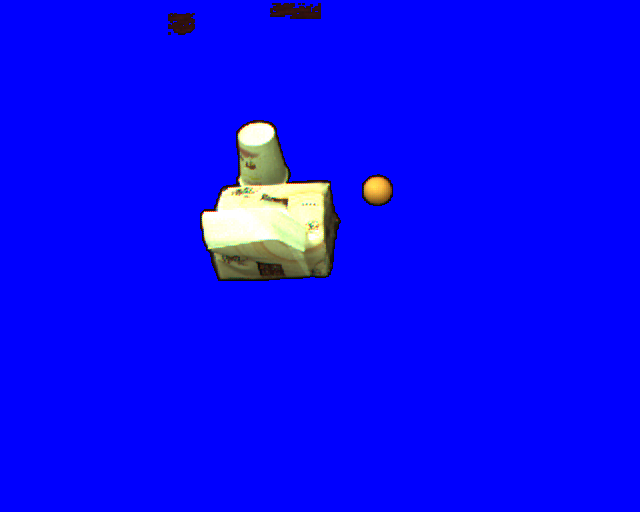

Supplement: S1 File — (ZIP) [file pone.0214852.s001.zip › 3D Reconstruction Experiment code/data/2_1.bmp]

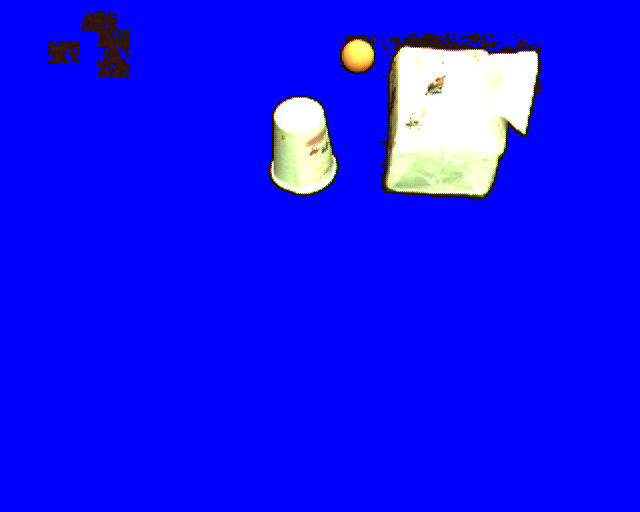

Supplement: S1 File — (ZIP) [file pone.0214852.s001.zip › 3D Reconstruction Experiment code/data/2_2.bmp]

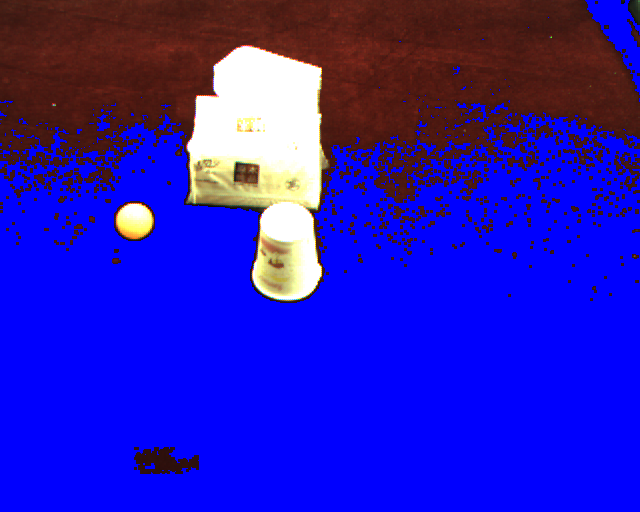

Supplement: S1 File — (ZIP) [file pone.0214852.s001.zip › 3D Reconstruction Experiment code/data/2_3.bmp]

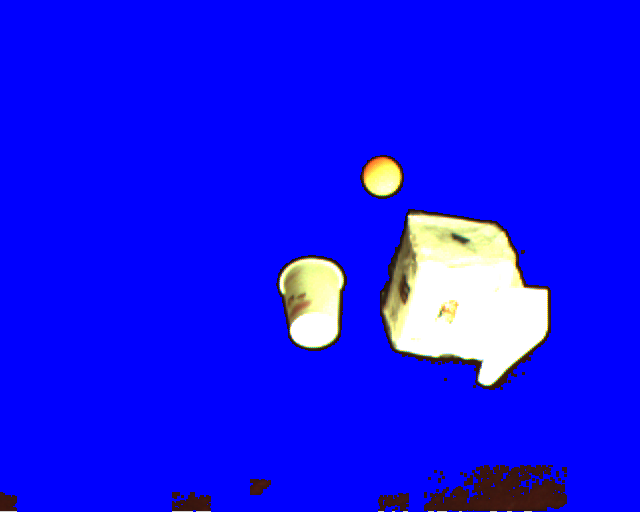

Supplement: S1 File — (ZIP) [file pone.0214852.s001.zip › 3D Reconstruction Experiment code/data/2_4.bmp]

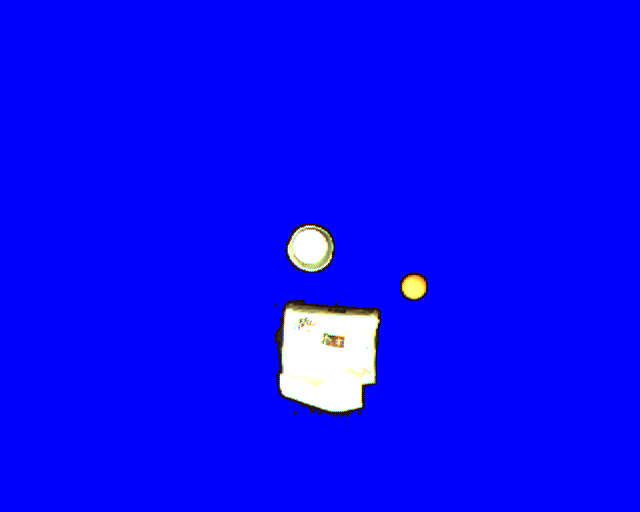

Supplement: S1 File — (ZIP) [file pone.0214852.s001.zip › 3D Reconstruction Experiment code/data/2_5.bmp]

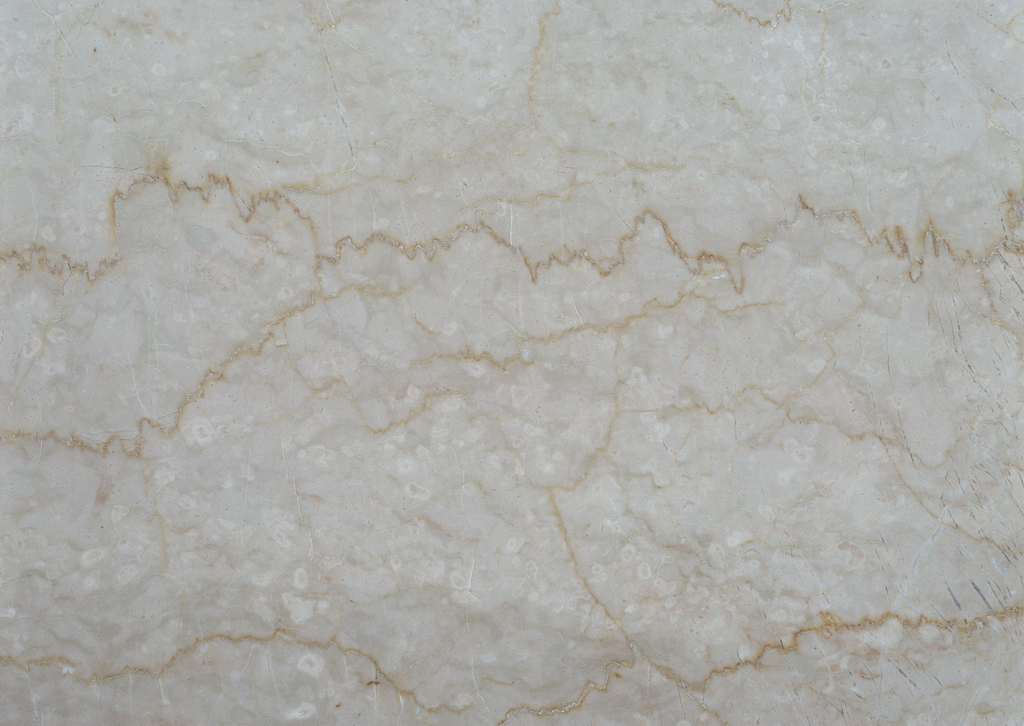

Supplement: S2 File — (ZIP) [file pone.0214852.s002.zip › Virtual Cutting Experiment code/cutting_simulation/Data/floor.bmp]

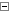

Supplement: S2 File — (ZIP) [file pone.0214852.s002.zip › Virtual Cutting Experiment code/_UpgradeReport_Files/UpgradeReport_Minus.gif]

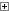

Supplement: S2 File — (ZIP) [file pone.0214852.s002.zip › Virtual Cutting Experiment code/_UpgradeReport_Files/UpgradeReport_Plus.gif]
